# Supplementary material for: Spatially mapped single-cell chromatin accessibility
Source: Nat Commun. 2021 Feb 24;12:1274. doi: 10.1038/s41467-021-21515-7 (PMC7904839; doi:10.1038/s41467-021-21515-7)
Supplement: Supplementary file 1 — Supplementary Information [file 41467_2021_21515_MOESM1_ESM.pdf]

Supplementary Information

Spatially mapped single-cell chromatin accessibility

Casey A. Thornton[1], Ryan M. Mulqueen[1], Kristof A. Torkenczy[1], Andrew Nishida[1], Eve G. Lowenstein[1], Andrew J. Fields[1], Frank J. Steemers[2], Wenri Zhang[3], Heather L. McConnell[4], Randy L. Woltjer[5], Anusha Mishra[4,6], Kevin M. Wright[7], Andrew C. Adey[1,6,8,9,*]

1) Molecular and Medical Genetics, Oregon Health & Science University, Portland, OR.
2) Illumina Inc. San Diego, CA
3) Anesthesiology and Peri-Operative Medicine, Oregon Health & Science University, Portland, OR.
4) Jungers Center for Neurosciences Research, Department of Neurology, Oregon Health & Science University, Portland, OR.
5) Department of Pathology, Oregon Health & Science University, Portland, OR.
6) Knight Cardiovascular Institute, Oregon Health & Science University, Portland, OR.
7) The Vollum Institute, Oregon Health & Science University, Portland, OR.
8) CEDAR, Oregon Health & Science University, Portland, OR.
9) Knight Cancer Institute, Oregon Health & Science University, Portland, OR.

* To whom correspondence should be addressed: adey@ohsu.edu

Supplementary Information includes Supplementary Figure 1-5

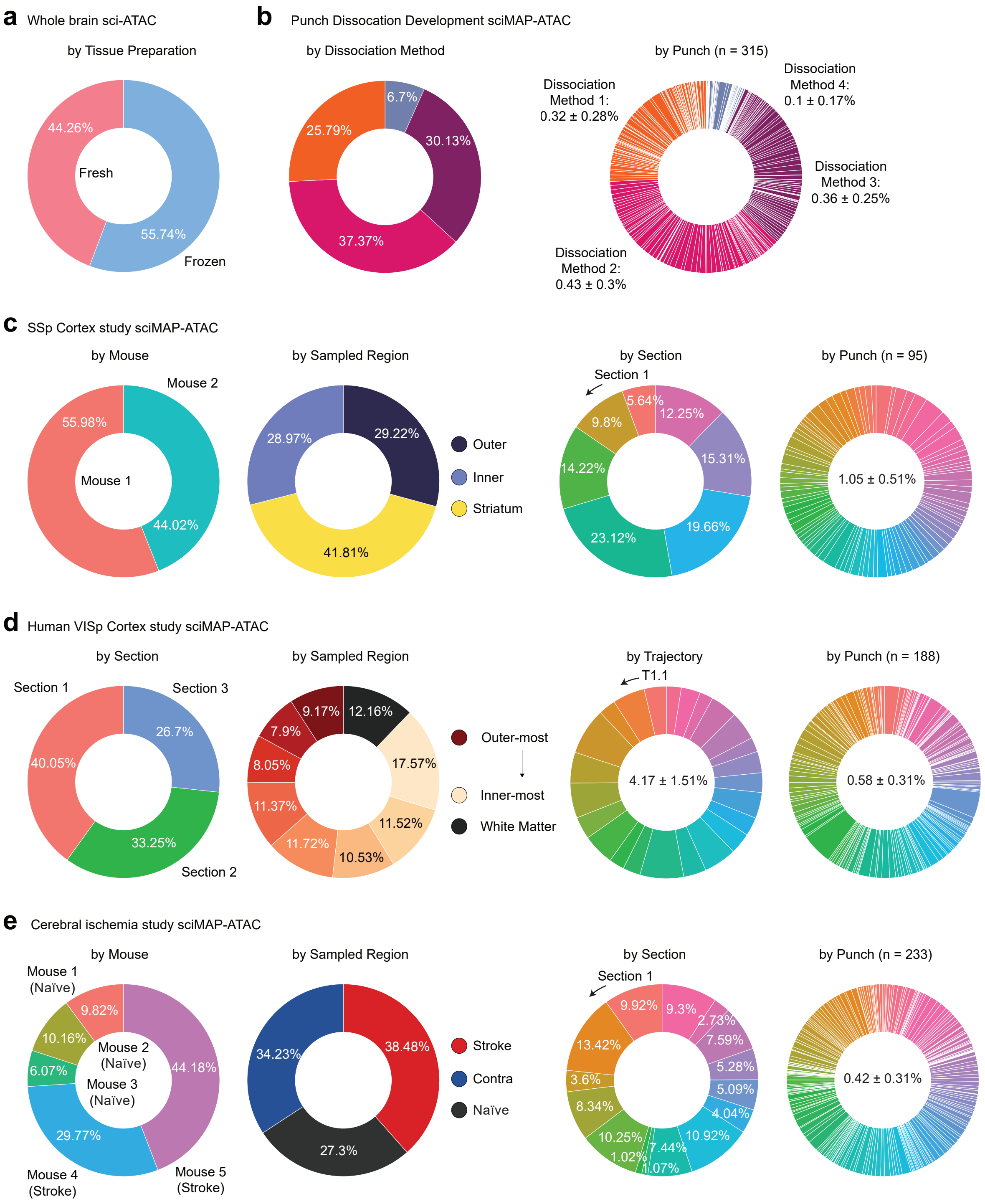

Supplementary Figure 1 | Overview of single-cell ATAC profiles produced across experimental conditions. Quality-passing single-cell ATAC-seq profiles for each experimental condition or spatially resolved punch (for sciMAP-ATAC) as a percentage of the experiment (or mean ± SD) for: **a.** sci-ATAC-seq on fresh vs. frozen mouse whole brain hemisphere; **b.** sciMAP-ATAC development across four dissociation methods and punches; colored by each dissociation method (n = 315 individual punches); **c.** sciMAP-ATAC on mouse SSp by biological replicate, class of sampled region, individual section, and punch (n = 95 individual punches).; **d.** Human VISp sciMAP-ATAC by section, position of sampled region, individual trajectory (T) and punch (n = 188 individual punches); and **e.** sciMAP-ATAC on a mouse model of cerebral ischemia by biological replicate, class of sampled region, section and punch (n = 233 individual punches).

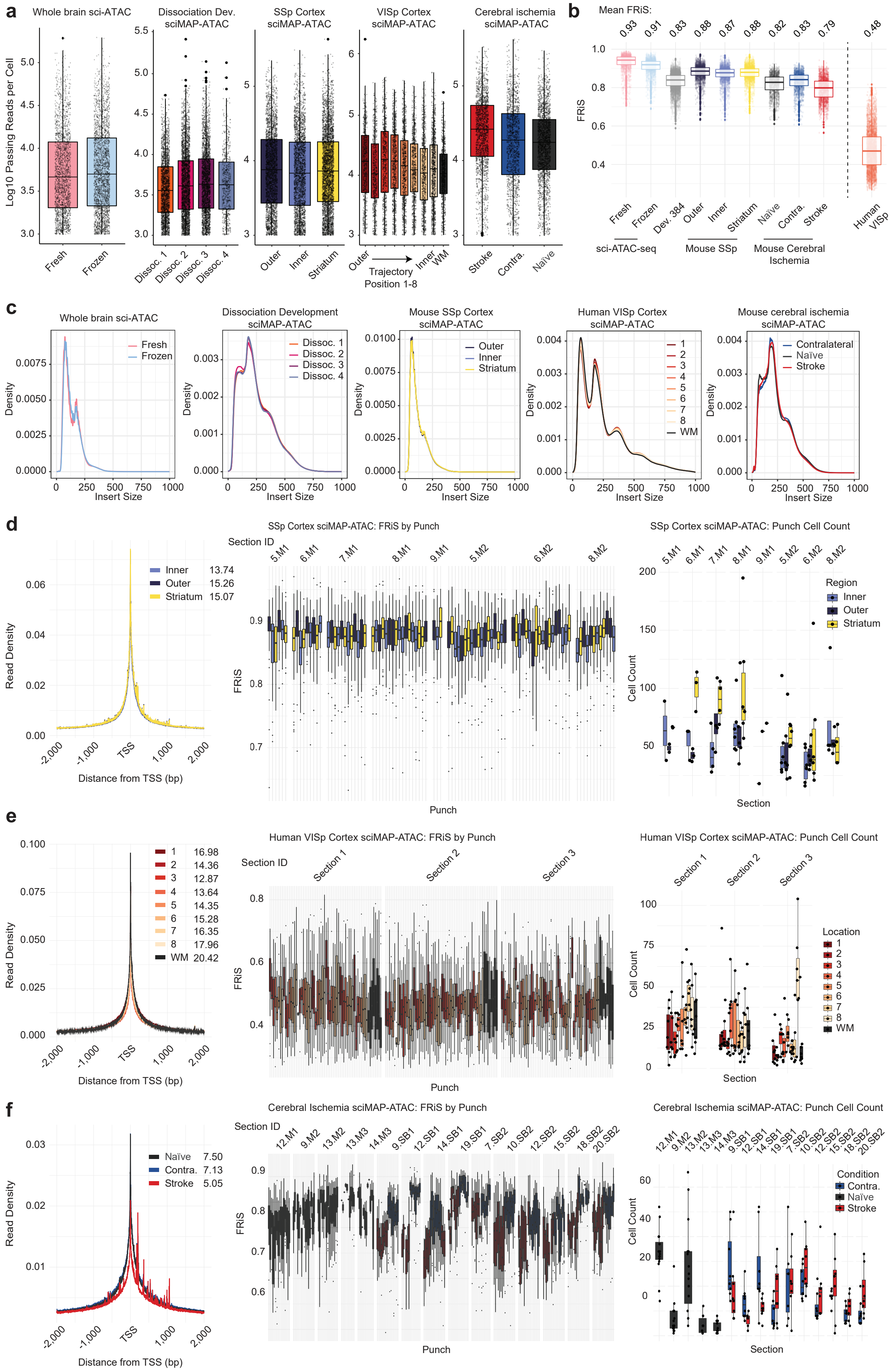
a
Whole brain sci-ATAC
Dissociation Dev. sciMAP-ATAC
SSp Cortex sciMAP-ATAC
VISp Cortex sciMAP-ATAC
Cerebral ischemia sciMAP-ATAC
Log10 Passing Reads per Cell
Fresh
Frozen
Dissoc. 1
Dissoc. 2
Dissoc. 3
Dissoc. 4
Outer
Inner
Striatum
Trajectory Position 1-8
WM
Stroke
Contra.
Naïve
b
Mean FRiS:
0.93 0.91 0.83 0.88 0.87 0.88 0.82 0.83 0.79 0.48
FRiS
Fresh
Frozen
Dev. 384
Outer
Inner
Striatum
Naïve
Contra.
Stroke
Human VISp
sci-ATAC-seq
Mouse SSp
Mouse Cerebral Ischemia
c
Whole brain sci-ATAC
Dissociation Development sciMAP-ATAC
Mouse SSp Cortex sciMAP-ATAC
Human VISp Cortex sciMAP-ATAC
Mouse cerebral ischemia sciMAP-ATAC
Density
Insert Size
Contralateral
Naïve
Stroke
d
Inner 13.74
Outer 15.26
Striatum 15.07
Read Density
Distance from TSS (bp)
SSp Cortex sciMAP-ATAC: FRiS by Punch
Section ID
5.M1 6.M1 7.M1 8.M1 9.M1 5.M2 6.M2 8.M2
Punch
SSp Cortex sciMAP-ATAC: Punch Cell Count
Region
Cell Count
Section
e
1 16.98
2 14.36
3 12.87
4 13.64
5 14.35
6 15.28
7 16.35
8 17.96
WM 20.42
Human VISp Cortex sciMAP-ATAC: FRiS by Punch
Section 1
Section 2
Section 3
Human VISp Cortex sciMAP-ATAC: Punch Cell Count
Location
f
Naïve 7.50
Contra. 7.13
Stroke 5.05
Cerebral Ischemia sciMAP-ATAC: FRiS by Punch
12.M1 9.M2 13.M2 13.M3 14.M3 9.SB1 12.SB1 14.SB1 19.SB1 7.SB2 10.SB2 12.SB2 15.SB2 18.SB2 20.SB2
Cerebral Ischemia sciMAP-ATAC: Punch Cell Count
Condition

Supplementary Figure 2 | Quality metrics across all experiments. a. Log10 passing reads obtained per cell at the depth of sequencing for all experiments: mouse whole brain sciATAC (n = 4,569 cells), mouse dissociation development sciMAP-ATAC (n = 8,011 cells), mouse SSp cortex sciMAP-ATAC (n = 7,779 cells), mouse VISp cortex sciMAP-ATAC (n = 4,547 cells), and mouse cerebral ischemia sciMAP-ATAC (n = 5,081 cells); as described in Supplementary Figure 1. Center line represents median, lower and upper hinges represent first and third quartiles, whiskers extend from hinge to ± 1.5x IQR, individual cells represented as dots. **b.** The fraction of reads present in a reference set of peaks (FRiS) for all cells in each experiment as in a. The master list of peaks for mouse are aggregated from ATAC-seq data produced by the ENCODE project, and for human it is from a single study on DNAse hypersensitivity[28]. Center line represents median, lower and upper hinges represent first and third quartiles, whiskers extend from hinge to ± 1.5x IQR, individual cells represented as colored dots. **c.** Insert size distributions for all experiments. **d-f.** Left: aggregate read density at transcription start sites (TSSs) and surrounding base pairs (bps) present in the genome with TSS enrichment values listed by each class calculated using the ENCODE method; middle: FRiS distributions for all cells within each punch produced in the experiment split by section and mouse cerebral ischemia sciMAP-ATAC (); and right: Punch distributions of cell counts for each category within the experiment split by section, for mouse SSp (d, n = 7,779 cells examined over 95 independent punches taken from 8 sections), human VISp (e, n = 4,547 cells examined over 188 independent punches taken from 3 sections) and mouse cerebral ischemia (f, n = 5,081 cells examined over 233 independent punches taken from 15 sections) experiments. Center line represents median, lower and upper hinges represent first and third quartiles, whiskers extend from hinge to ± 1.5x IQR, individual cells represented as dots.

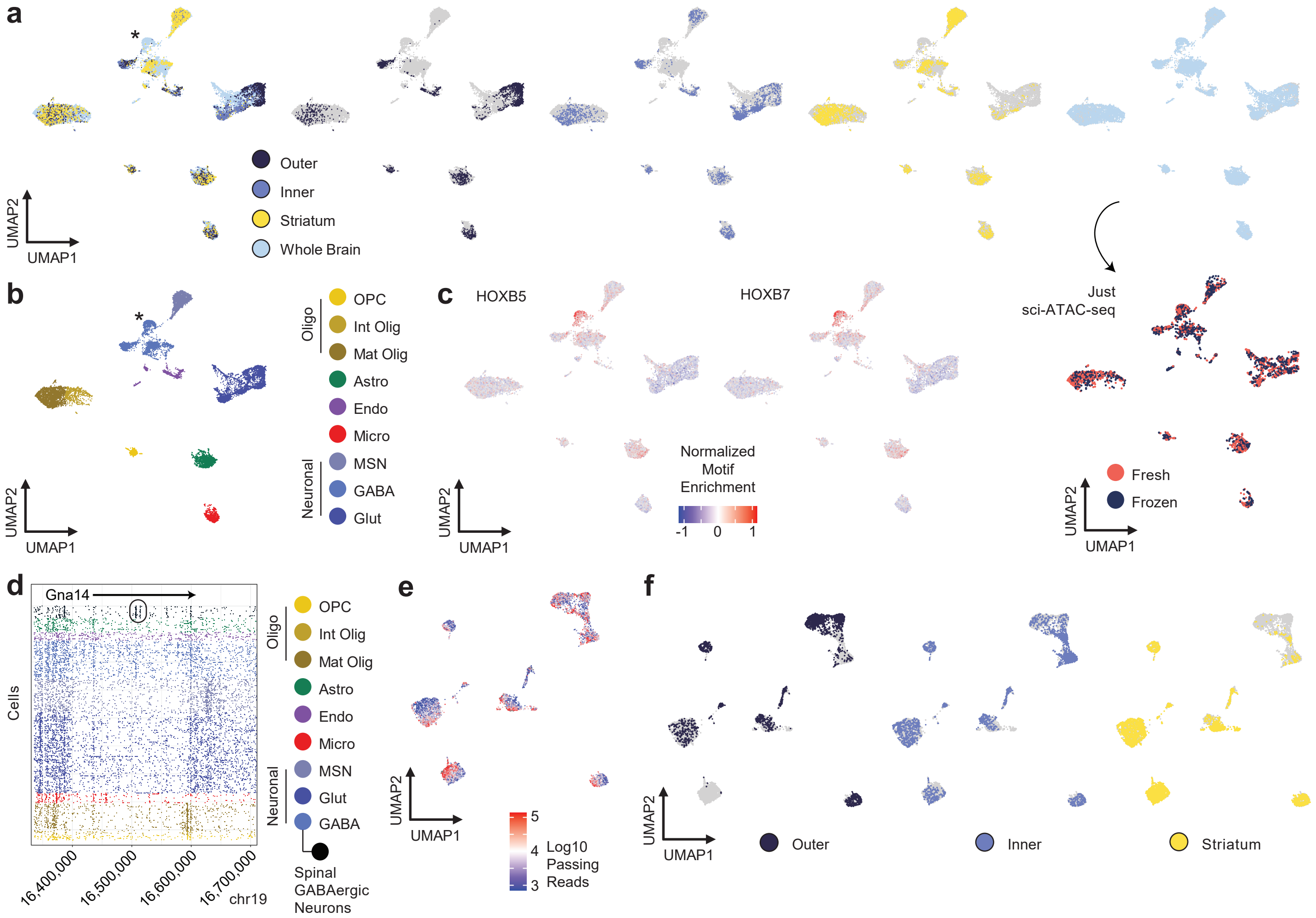

Supplementary Figure 3 | Extended analysis of the mouse somatosensory cortex sciMAP-ATAC dataset. a. Integration of all healthy mouse brain sci-ATAC-seq and sciMAP-ATAC datasets visualized in a UMAP. From left to right: all cells colored bythe regional category of punch position (outer cortex, inner cortex, striatum) for the SSp experiment and then cells from whole brain experiments (sciMAP-ATAC and sci-ATAC-seq).Asterisk indicates the population of cells only present in the whole brain dataset. Cells are grayed out except for those from punches taken from the outer cortex, inner cortex, stratum and then whole brain. Below the whole brain panel, cells derived from the sci-ATAC-seq experiment on fresh and frozen brain hemispheres are indicated. **b.** The same integrated UMAP with cells colored by identified cell type, as defined in Figure 2d. Asterisk indicates the population of GABAergic neurons only present in whole brain datasets that represent spinal cord derived interneurons. **c.** HOXB5 and HOXB7 are two example motifs that exhibit increased accessibility in the spinal cord derived interneuron population. **d.** ATAC reads for cells (rows) are shown for the *Gna14* locus with cells colored by cell type, as defined in Figure 2d, with the addition of the spinal GABAergic neuron subcluster. The cluster representing spinal cord derived interneurons is shown in black with the uniquely accessible loci circled. **e.** UMAP of the SSp dataset with cells colored by log10 passing read counts. **f.** UMAP of the SSp dataset with cells grayed out except for each of the three regional punch categories; outer cortex, inner cortex, and striatum.

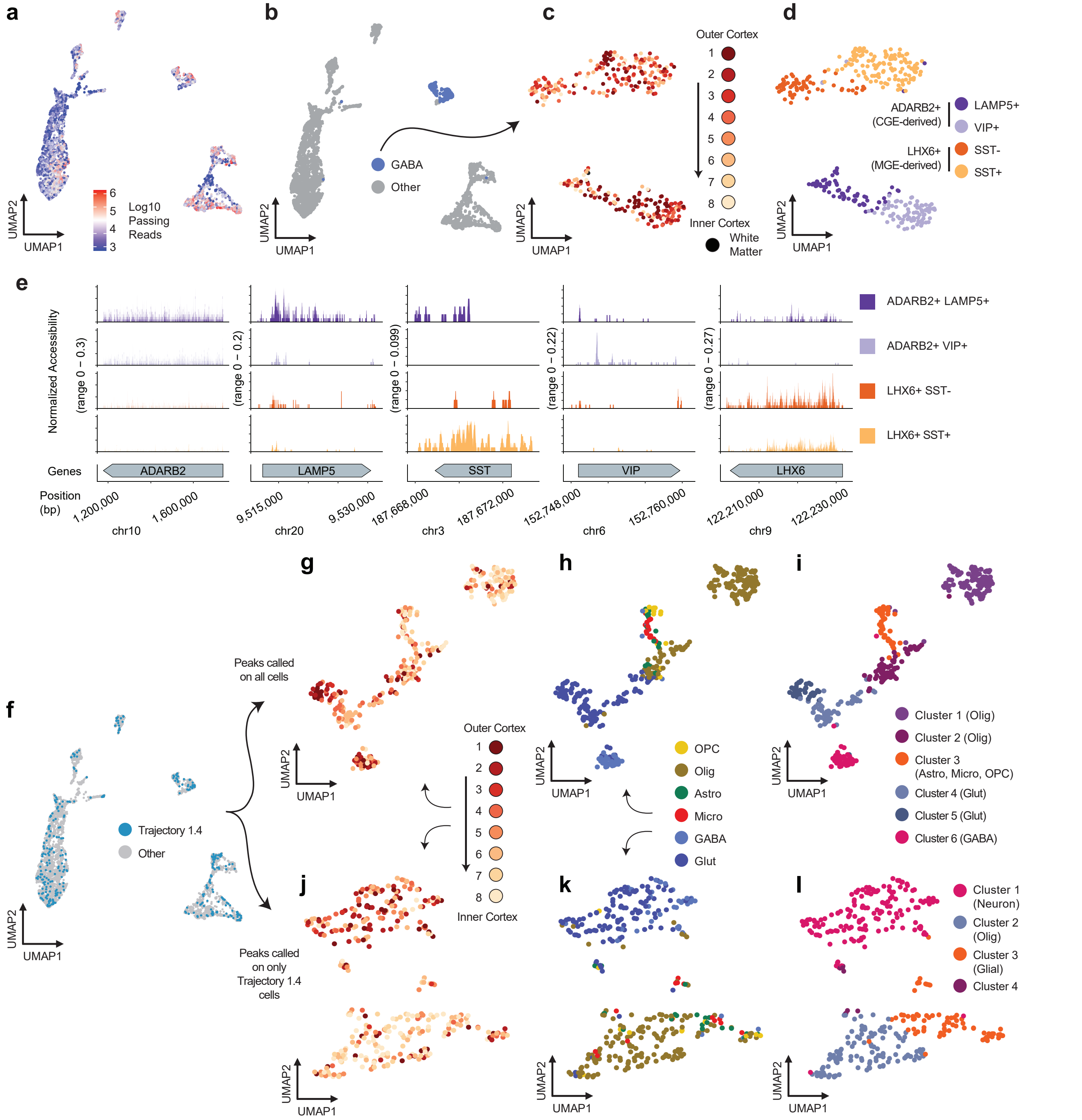

Supplementary Figure 4 | Extended analysis of the human primary visual cortex sciMAP-ATAC dataset. **a.** UMAP of all cells from the experiment colored by log10 passing read counts. **b.** UMAP of the full dataset with all cells grayed out except for those identified as GABAergic neurons. **c.** UMAP of GABAergic neurons analyzed using topic modeling individually colored by punch position. **d.** Four interneuron clusters identified, including two MGE-derived and two CGE-derived cell types. **e.** Aggregate ATAC-seq profiles for marker genes for each of the interneuron cell types. **f.** UMAP of the full dataset with all cells grayed out except for those belonging to the fourth trajectory, of 8 consecutive punches, on the first section (Trajectory 1.4). **g.** UMAP of cells from Trajectory 1.4 that were processed using peaks from the full VISp dataset colored by the punch position; **h.** the cell type classification as determined from the full dataset; and **i.** the six clusters that were identified. **j.** UMAP of cells from Trajectory 1.4 that were processed using peaks called using only those cells, colored by the punch position; **k.** the cell type classification as determined from the full dataset; and **i.** the four clusters that were identified.

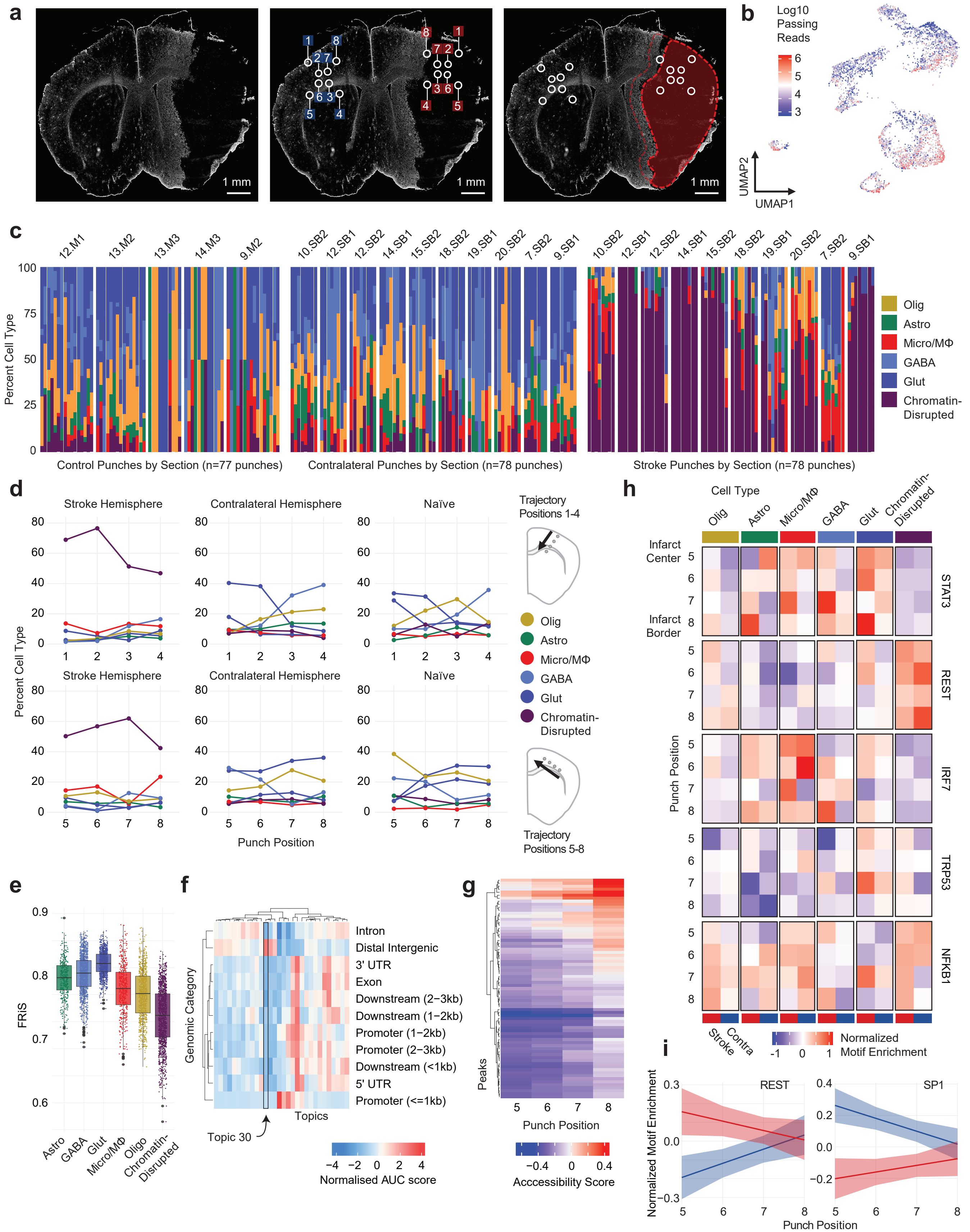

a
1 mm
b
Log10 Passing Reads
6 5 4 3
UMAP2
UMAP1
c
12.M1 13.M2 13.M3 14.M3 9.M2
10.SB2 12.SB1 12.SB2 14.SB1 15.SB2 18.SB2 19.SB1 20.SB2 7.SB2 9.SB1
Percent Cell Type
Olig
Astro
Micro/MΦ
GABA
Glut
Chromatin-Disrupted
Control Punches by Section (n=77 punches)
Contralateral Punches by Section (n=78 punches)
Stroke Punches by Section (n=78 punches)
d
Stroke Hemisphere
Contralateral Hemisphere
Naïve
Trajectory Positions 1-4
Trajectory Positions 5-8
Percent Cell Type
Punch Position
e
FRiS
Astro GABA Glut Micro/MΦ Oligo Chromatin-Disrupted
f
Intron
Distal Intergenic
3' UTR
Exon
Downstream (2–3kb)
Downstream (1–2kb)
Promoter (1–2kb)
Promoter (2–3kb)
Downstream (<1kb)
5' UTR
Promoter (<=1kb)
Genomic Category
Topics
Topic 30
Normalised AUC score
g
Peaks
Punch Position
Acccessibility Score
h
Cell Type
Olig Astro Micro/MΦ GABA Glut Chromatin-Disrupted
Infarct Center
Infarct Border
Punch Position
STAT3
REST
IRF7
TRP53
NFKB1
Stroke Contra
Normalized Motif Enrichment
i
REST
SP1
Normalized Motif Enrichment
Punch Position

Supplementary Figure 5 | Extended analysis of the cerebral ischemia sciMAP-ATAC dataset. a. Representative GFAP immunostaining of a histoligcalsection from a stroke brain (left), with punch positions and labels shown (middle), and punch positions with the stroke region overlaid in red (right). **b.** UMAP of cells from the cerebral ischemia experiment colored by the log10 passing read counts. **c.** Cell type composition for each punch in the experiment grouped by individual section and more broadly by category; colored by cell type as defined in Figure 7d **d.** Aggregated cell type composition for the 1-4 axis (top) and 5-8 axis (bottom) split by category of tissue; colored by cell type as defined in Figure 7d. **e.** FRiS values for cells split by cell type indicating a substantial decrease in FRiS for the chromatin-disrupted cluster (n = 5,081 cells); colored by cell type as defined in Figure 7d. Center line represents median, lower and upper hinges represent first and third quartiles, whiskers extend from hinge to ± 1.5x IQR, individual cells represented as colored dots. **f.** Enrichment for topics with respect to genomic category showing that Topic 30, which is elevated in cells within the chromatin-disrupted cluster, is enriched for distal intergenic regions – further supporting a global laxing of chromatin, likely due to cell death. **g.** Regulatory elements that change significantly and uniformly along the 5-8 axis. *P* value of the two-way ANOVA from the interaction of regulatory element site enrichment per punch by condition without multiple comparison correction ($p < 0.05$). **h.** Motif enrichment along the 5-8 axis for stroke and contralateral hemispheres split by cell type as defined in Figure 7d. **i.** REST and SP1 normalized motif enrichment along the 5-8 axis shows opposite trends between the two factors as well as for each factor between the stroke and contra hemispheres. Data are presented as linear fitted model ± SEM. Source data are provided as a Source Data file.
