# Supplementary material for: Spatially mapped single-cell chromatin accessibility
Source: Nat Commun. 2021 Feb 24;12:1274. doi: 10.1038/s41467-021-21515-7 (PMC7904839; doi:10.1038/s41467-021-21515-7)
Supplement: Supplementary file 2 — Description of Additional Supplementary Files [file 41467_2021_21515_MOESM2_ESM.pdf]

Title: Supplementary Data 1
Description: Marker gene accessibility plots. ATAC read depth aggregated by identified cell types are plotted for cell type identification marker genes for the mouse SSp cortex, human VISp cortex, and mouse cerebral ischemia sciMAP-ATAC datasets.

Title: Supplementary Data 2
Description: UMAPs split by each individual punch or trajectory. UMAPs for the mouse SSp cortex and cerebral ischemia experiment are shown with all cells greyed out except for each indicated punch as well as UMAPs for the human VISp cortex experiment with all cells greyed out except for each individual punch trajectory.

Title: Supplementary Data 3
Description: Moran's I-test results for human VISp. Test results to determine significance of spatial contribution to epigenetic profiles for each cell type in the human VISp sciMAP-ATAC dataset (see Methods). P values of the unpaired one-sided z-test with Bonferroni-Holm correction ($p < 0.05$).

Title: Supplementary Data 4
Description: Interactive 3D UMAP of glutamatergic neurons from the human VISp. Cells identified as glutamatergic neurons embedded in a three-dimensional UMAP and colored by punch position.

Title: Supplementary Data 5
Description: Oligonucleotide Sequences. Primers and barcodes for single-cell combinatorial 96-plex sample indexing and custom sequencing.
