# Supplementary figures and images for: Spatially mapped single-cell chromatin accessibility
Source: Nat Commun. 2021 Feb 24;12:1274. doi: 10.1038/s41467-021-21515-7 (PMC7904839; doi:10.1038/s41467-021-21515-7)

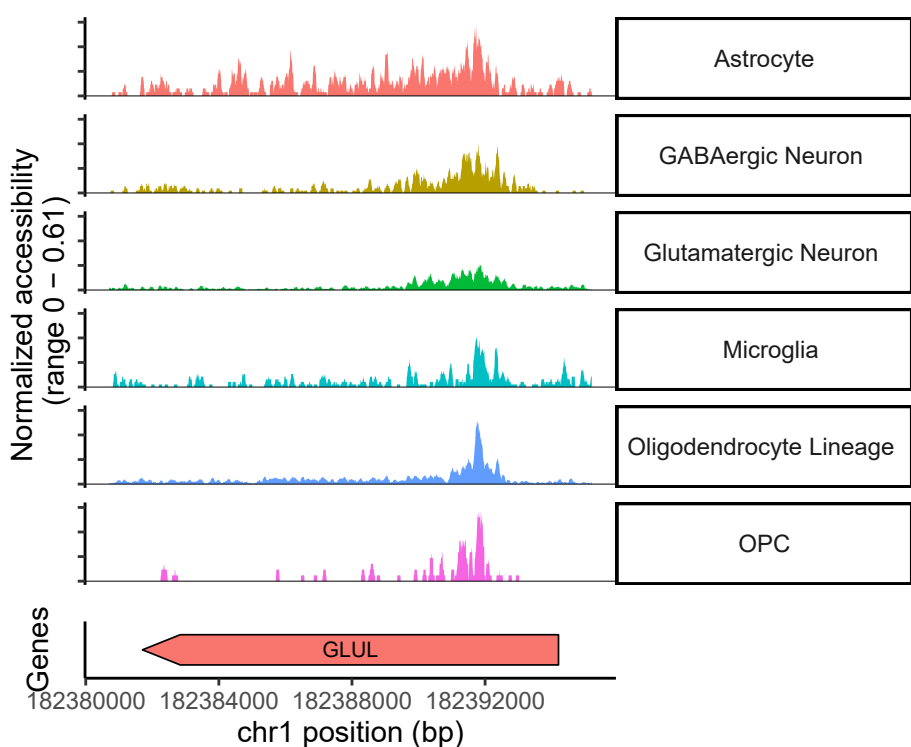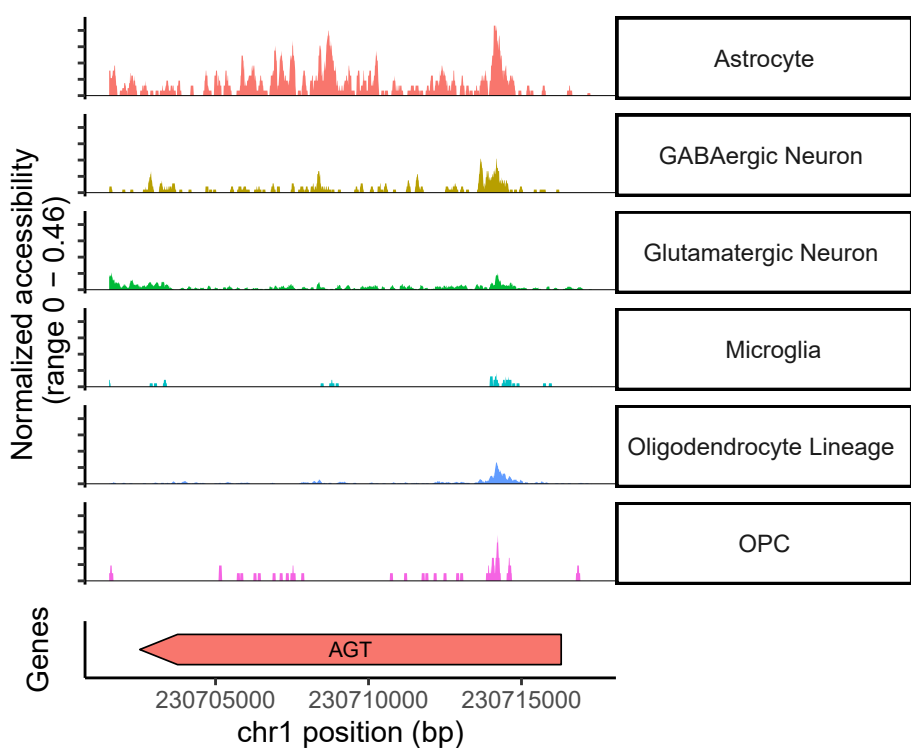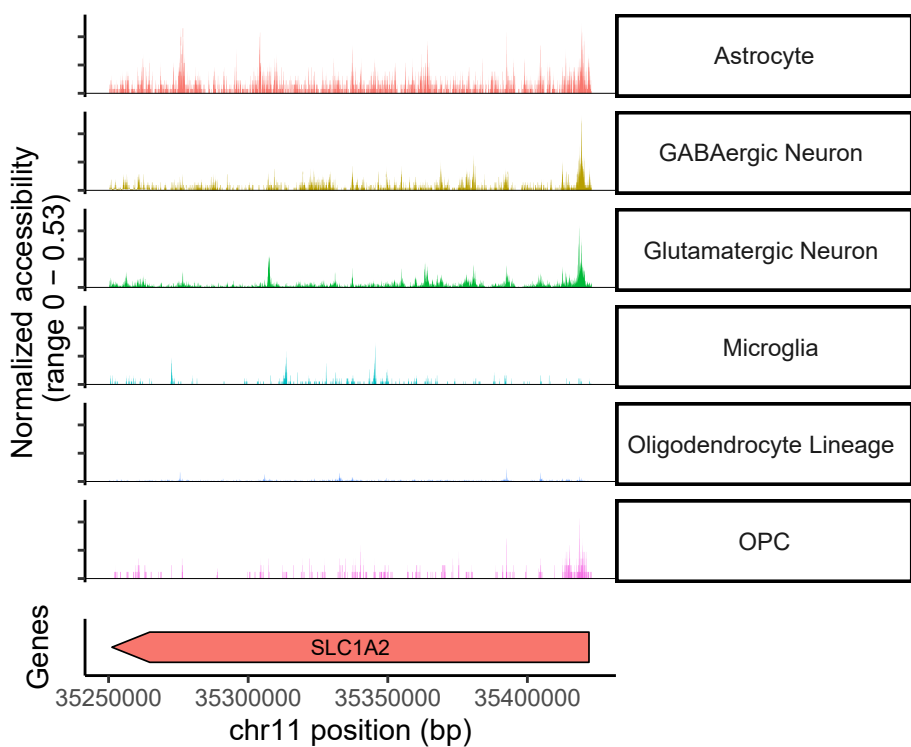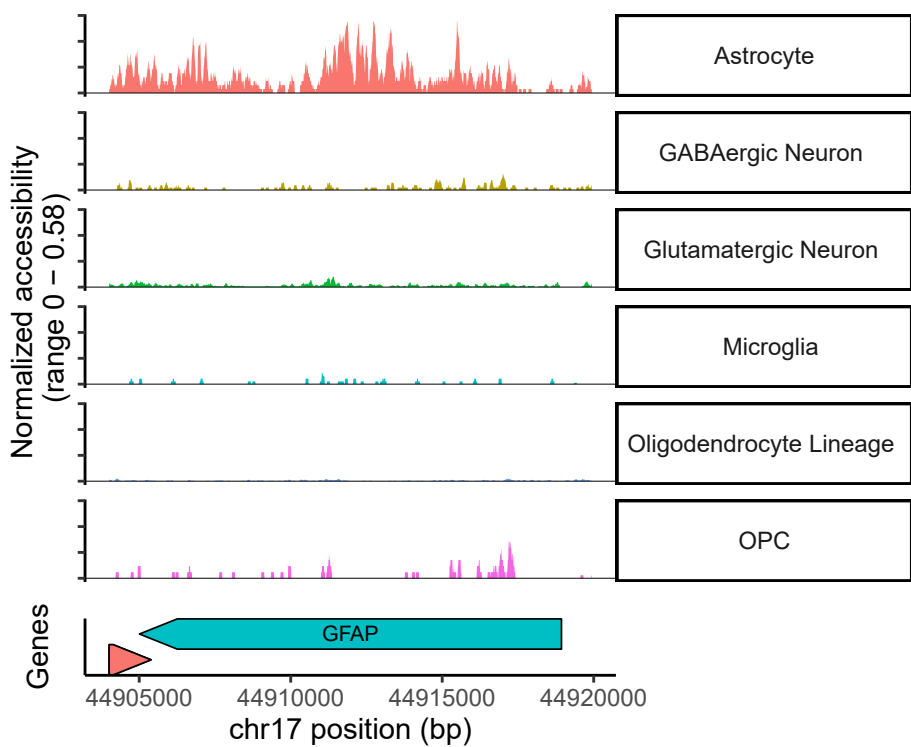

Supplement: Supplementary file 3 — Supplementary Data 1 [file 41467_2021_21515_MOESM3_ESM.zip › Corrected_CelltypeMarker_Plots/HumanVISpCortex.markeraccessibility/HumanVISpCortex.Astro.markeraccessibility.pdf]

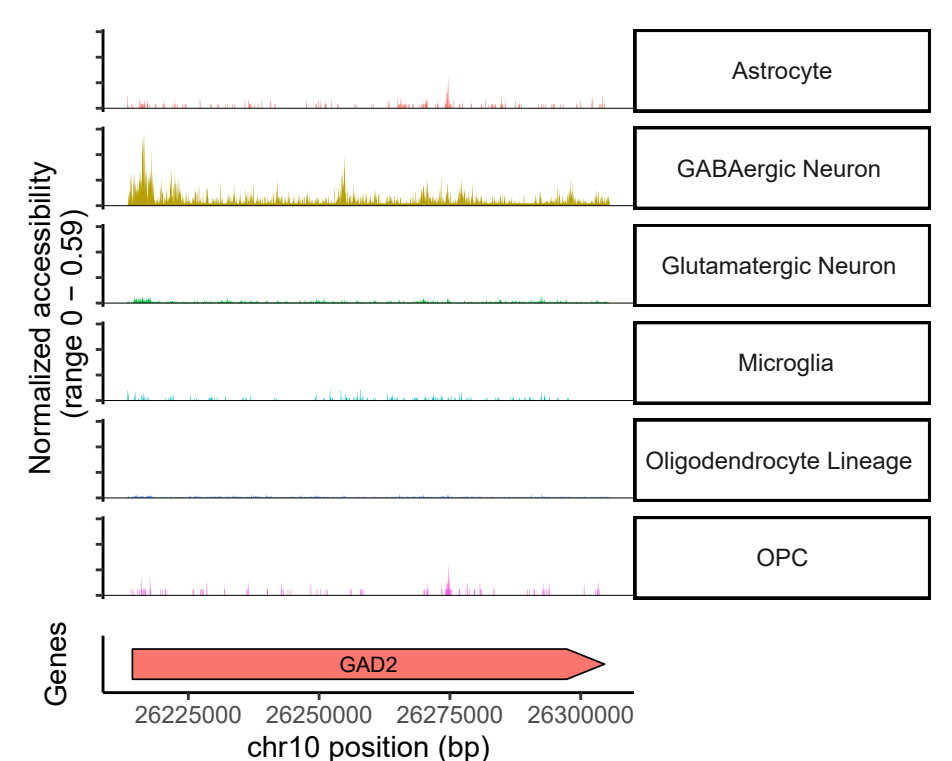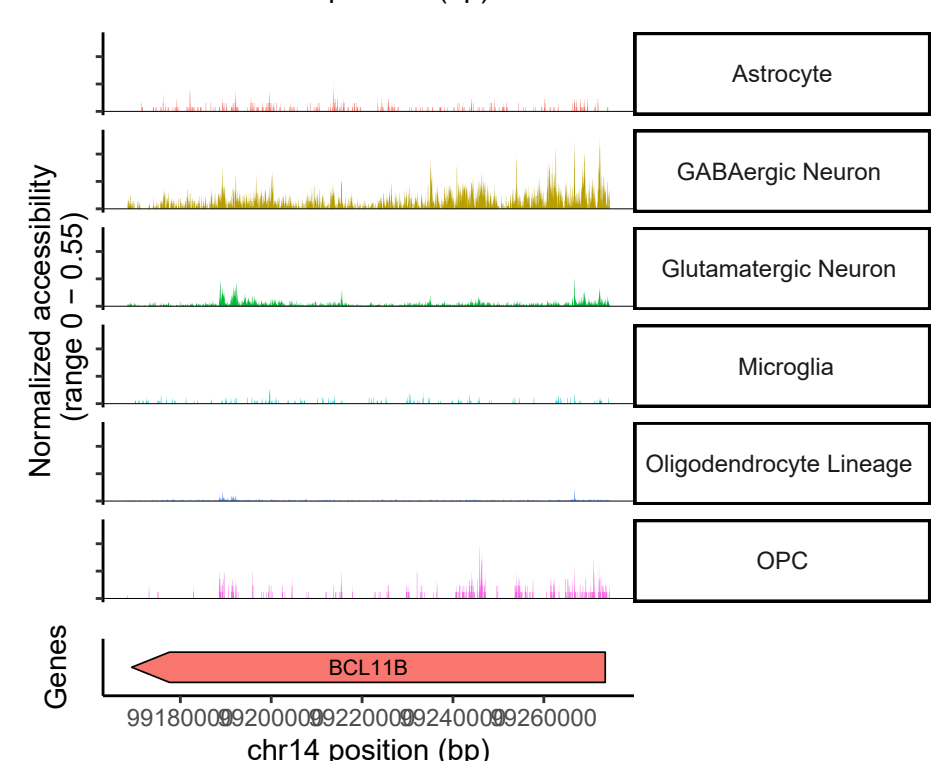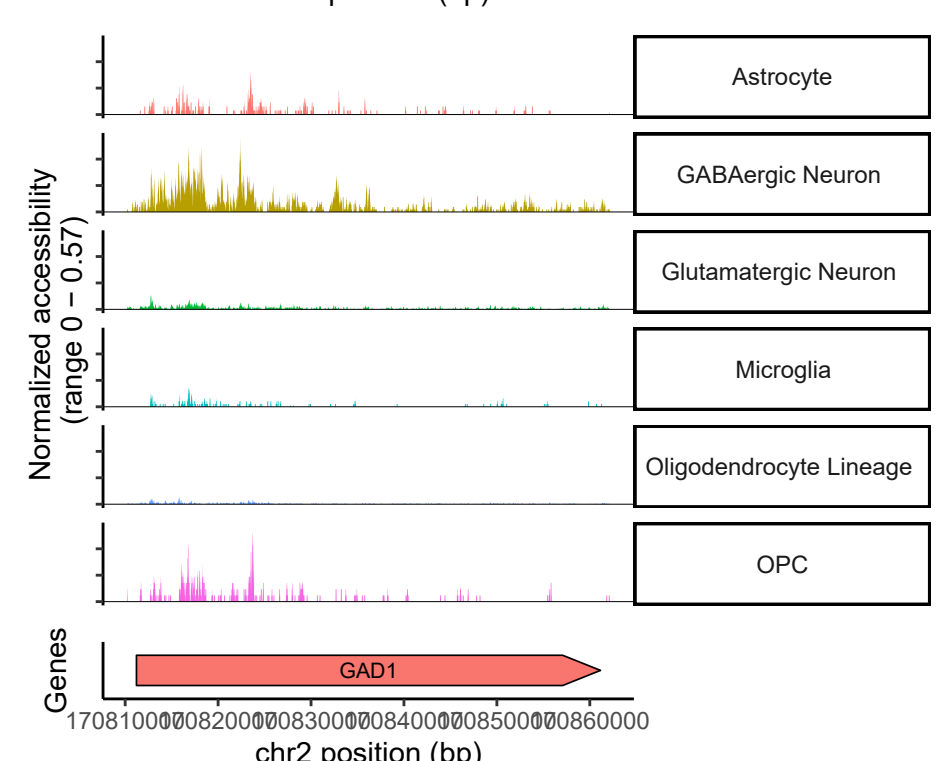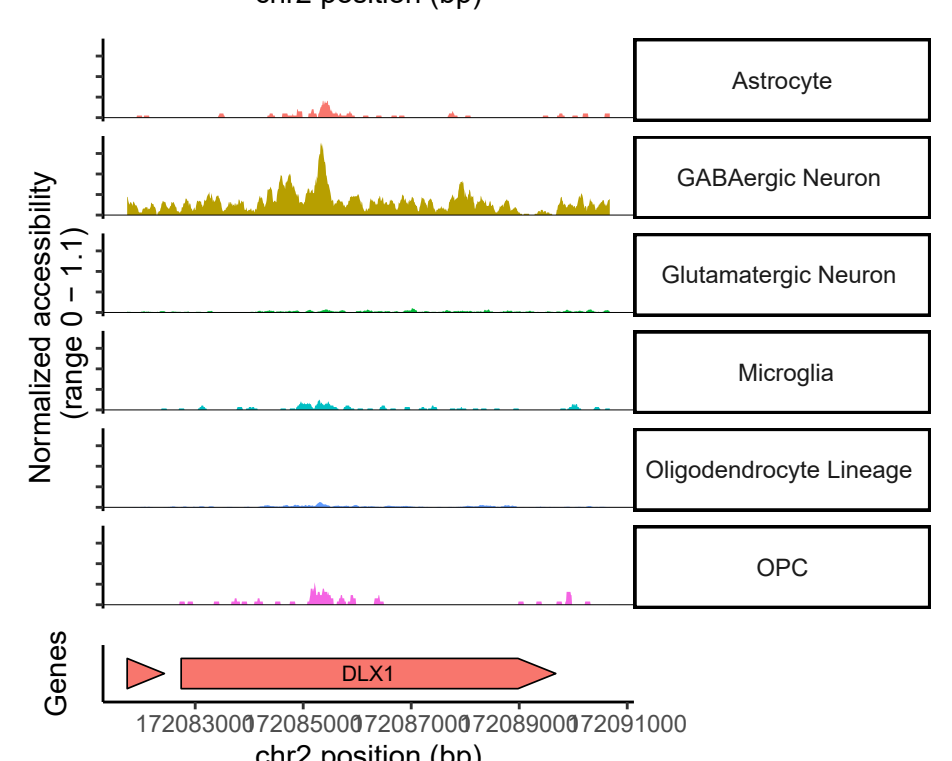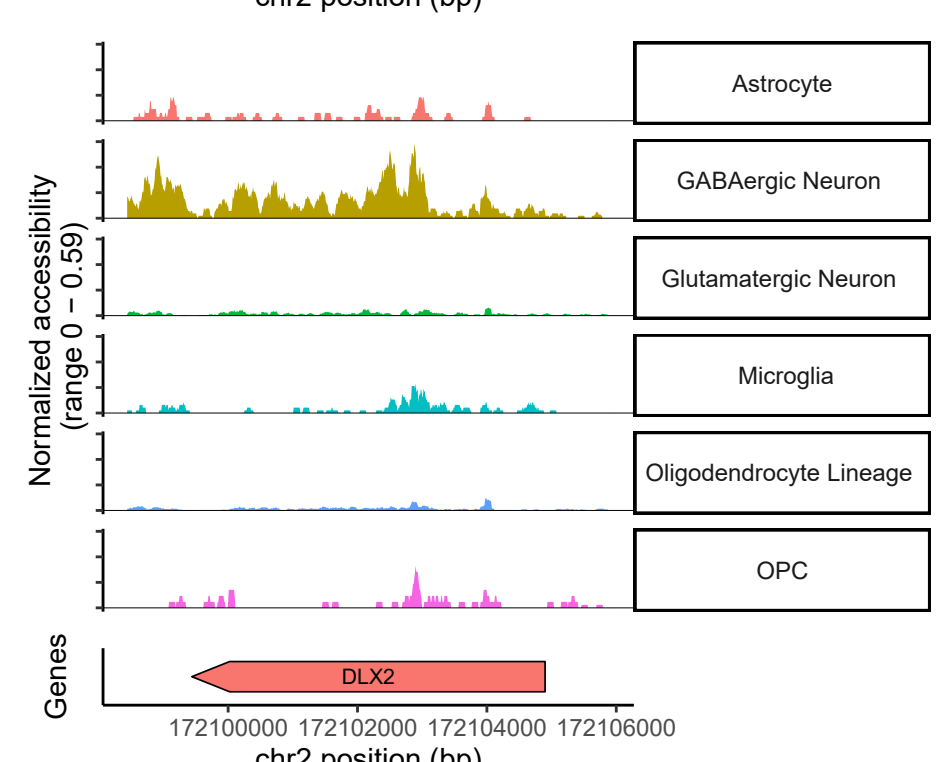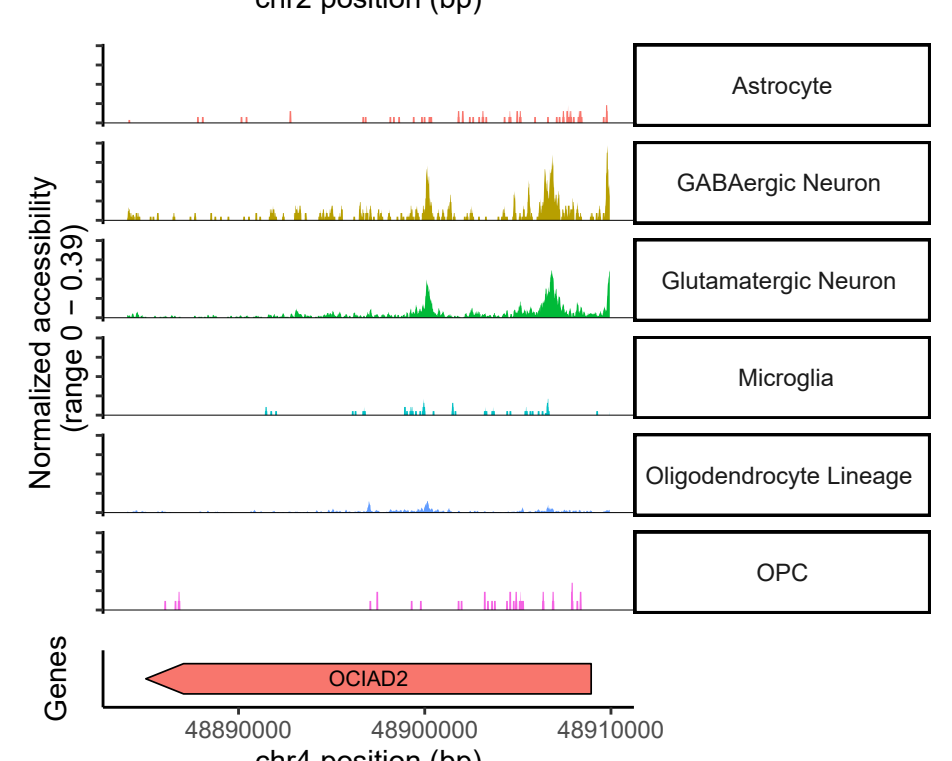

Supplement: Supplementary file 3 — Supplementary Data 1 [file 41467_2021_21515_MOESM3_ESM.zip › Corrected_CelltypeMarker_Plots/HumanVISpCortex.markeraccessibility/HumanVISpCortex.GABA.markeraccessibility.pdf]

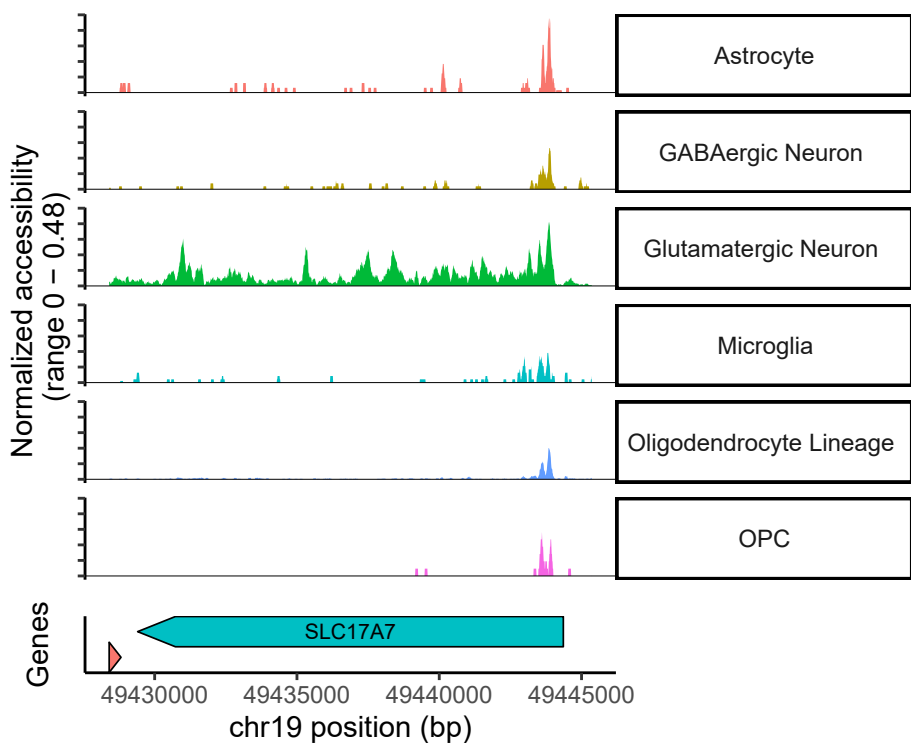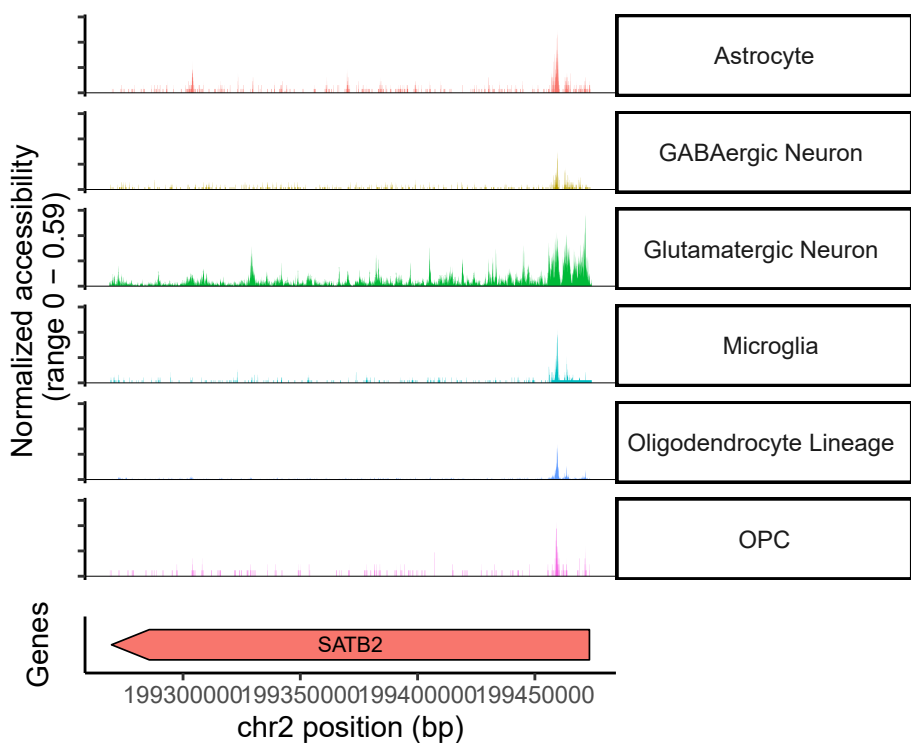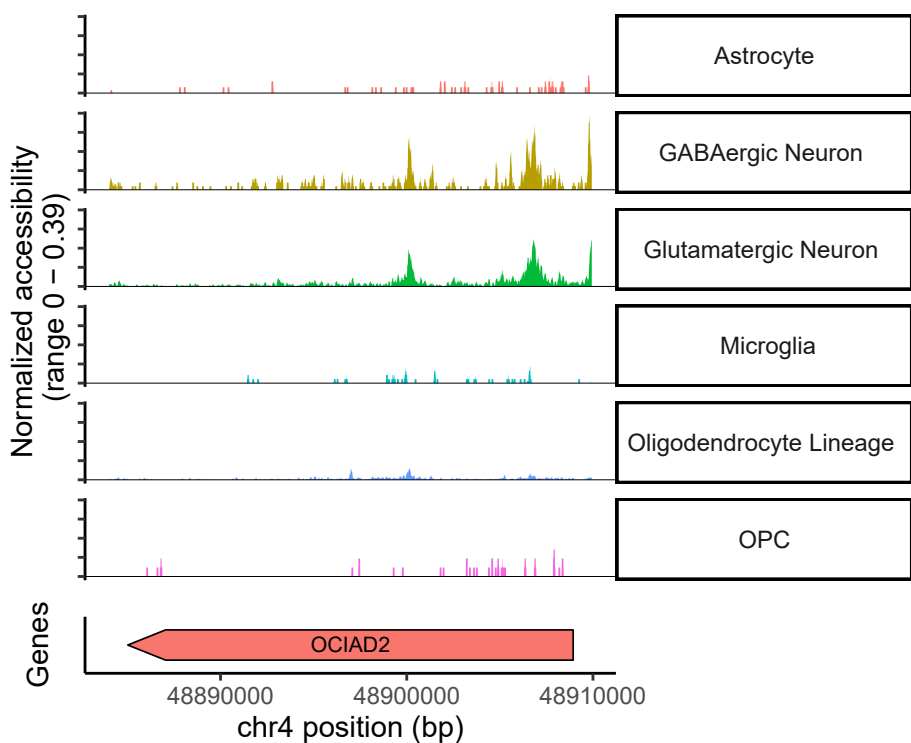

Supplement: Supplementary file 3 — Supplementary Data 1 [file 41467_2021_21515_MOESM3_ESM.zip › Corrected_CelltypeMarker_Plots/HumanVISpCortex.markeraccessibility/HumanVISpCortex.Glut.markeraccessibility.pdf]

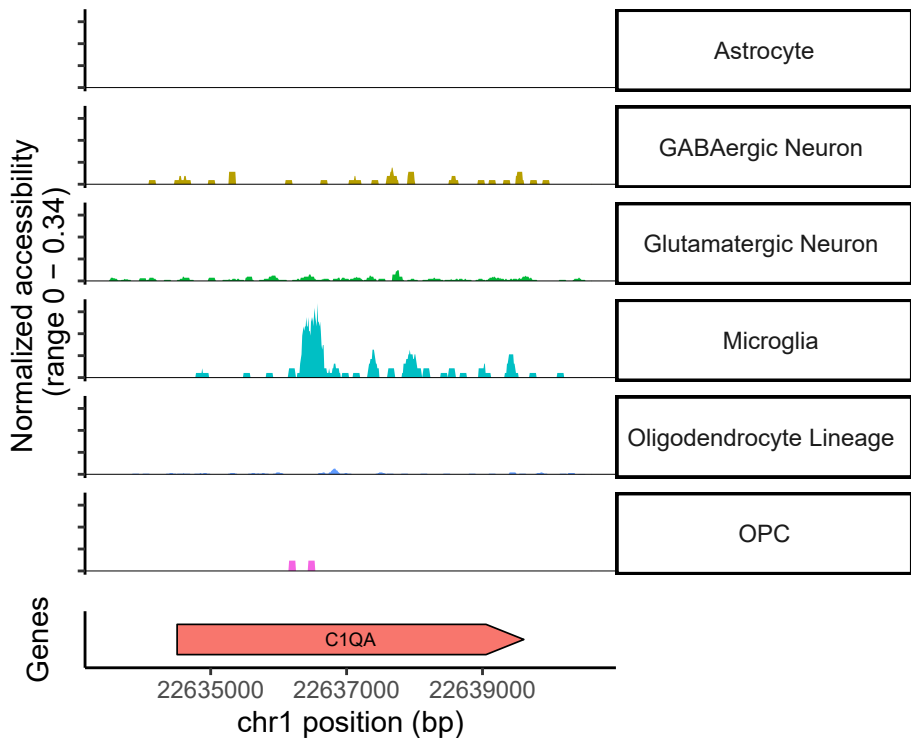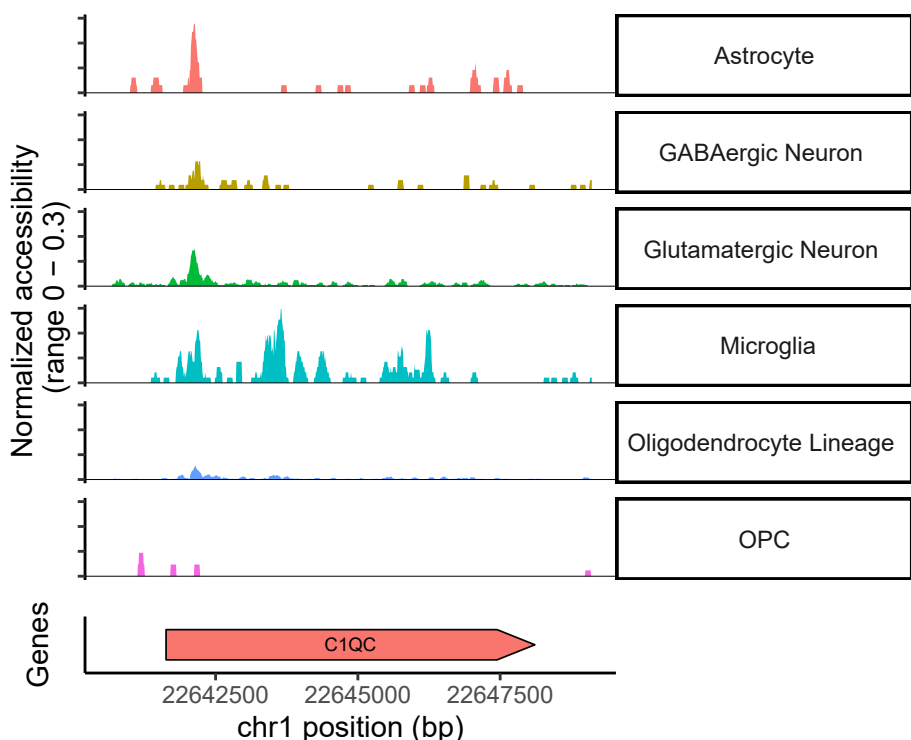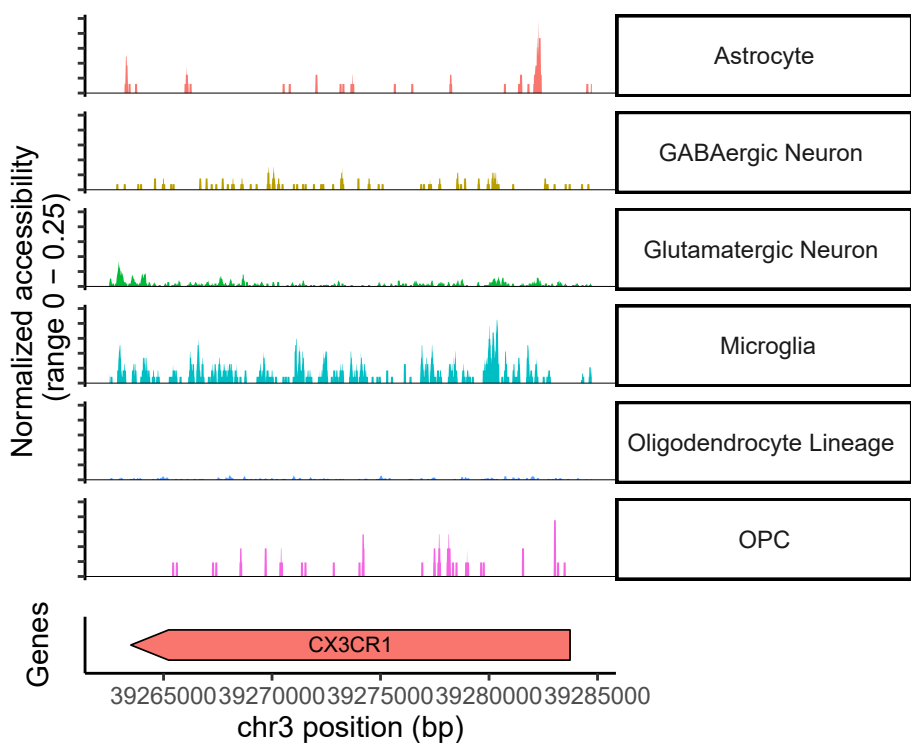

Supplement: Supplementary file 3 — Supplementary Data 1 [file 41467_2021_21515_MOESM3_ESM.zip › Corrected_CelltypeMarker_Plots/HumanVISpCortex.markeraccessibility/HumanVISpCortex.Micro.markeraccessibility.pdf]

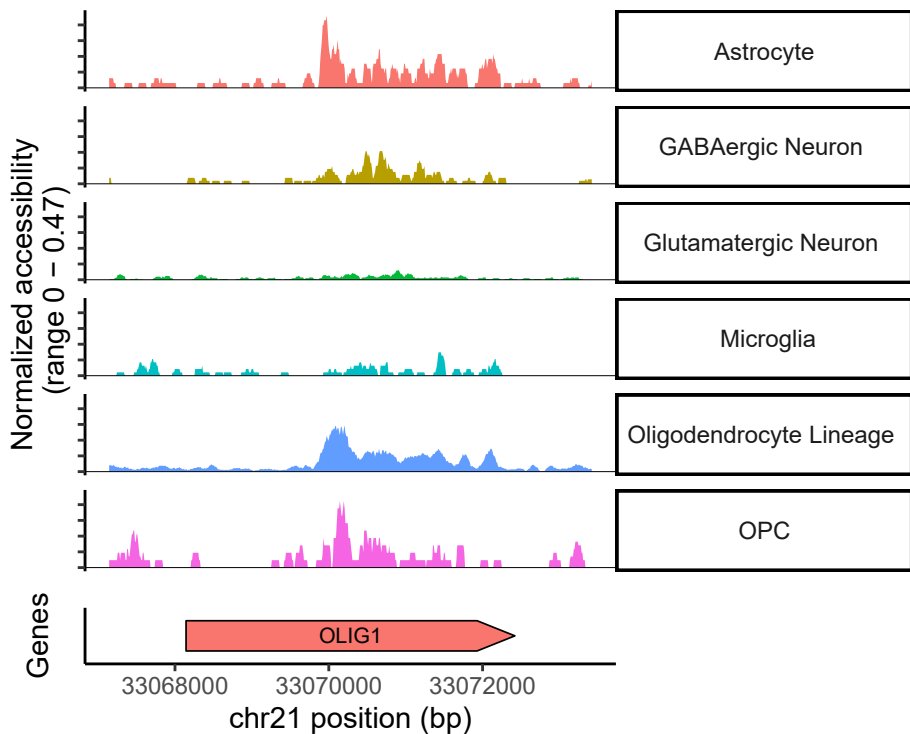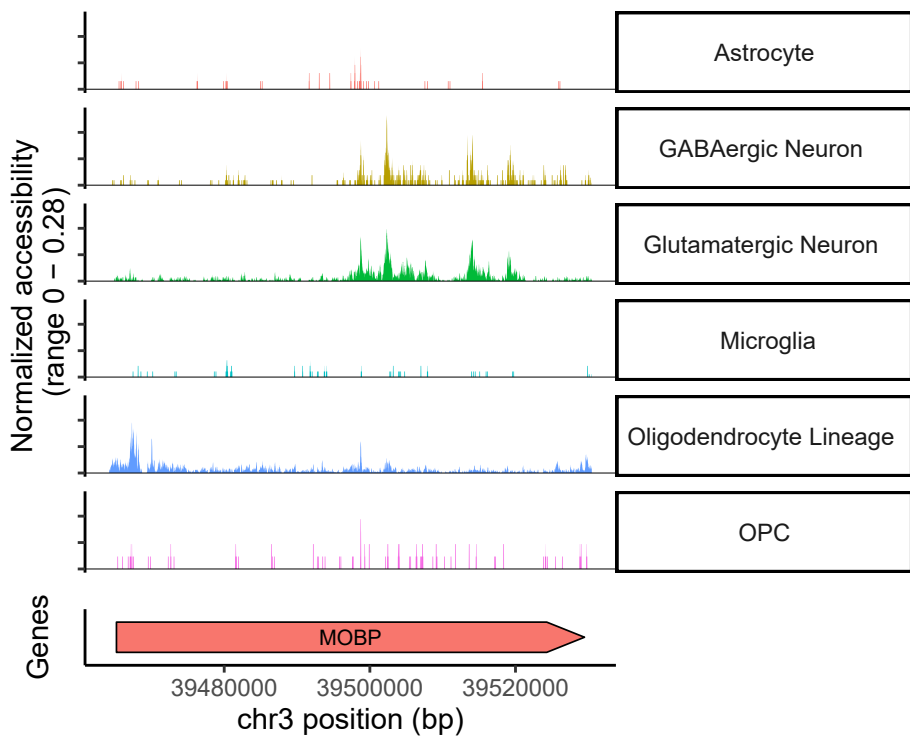

Supplement: Supplementary file 3 — Supplementary Data 1 [file 41467_2021_21515_MOESM3_ESM.zip › Corrected_CelltypeMarker_Plots/HumanVISpCortex.markeraccessibility/HumanVISpCortex.Olig.markeraccessibility.pdf]

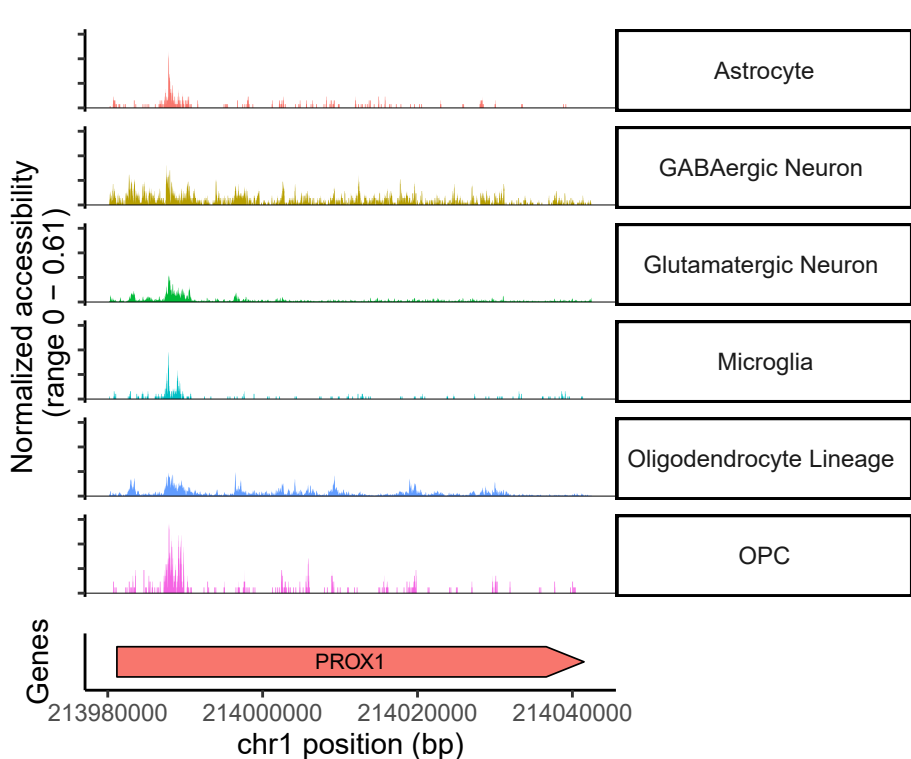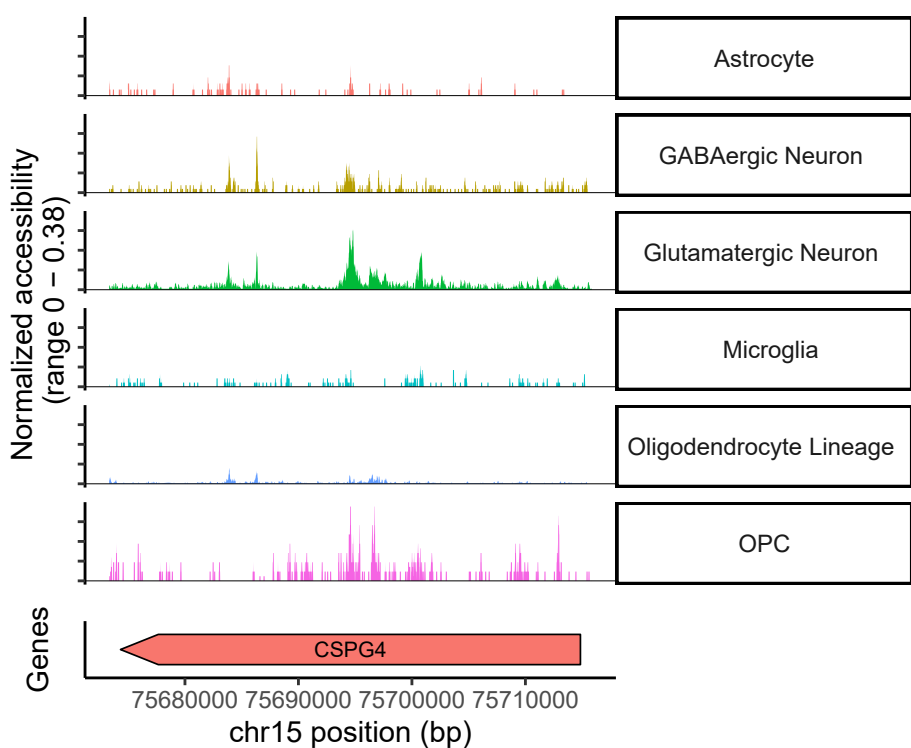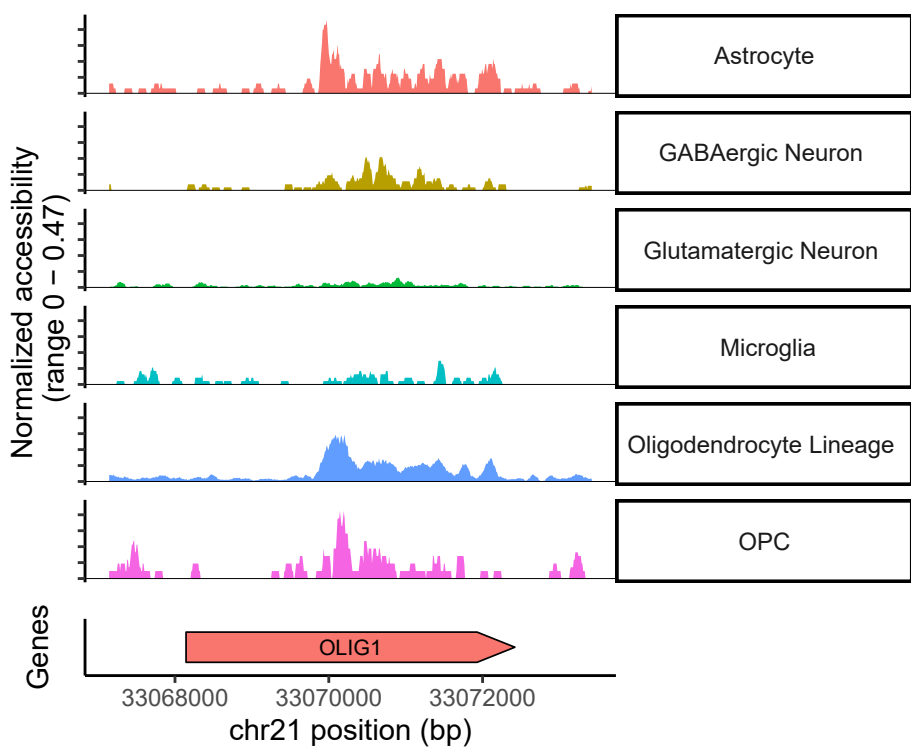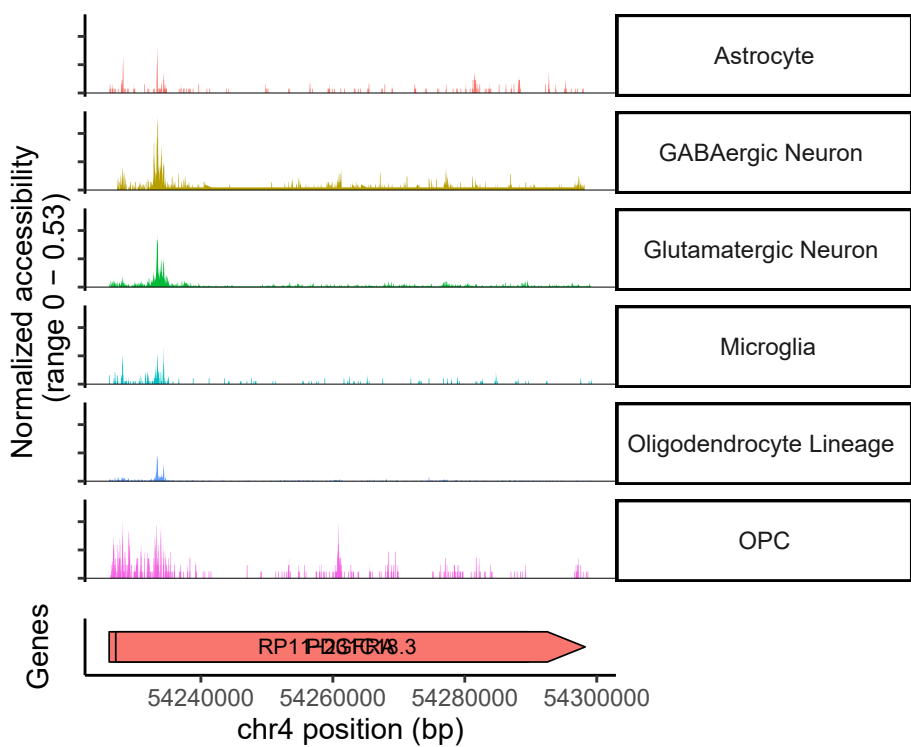

Supplement: Supplementary file 3 — Supplementary Data 1 [file 41467_2021_21515_MOESM3_ESM.zip › Corrected_CelltypeMarker_Plots/HumanVISpCortex.markeraccessibility/HumanVISpCortex.OPC.markeraccessibility.pdf]

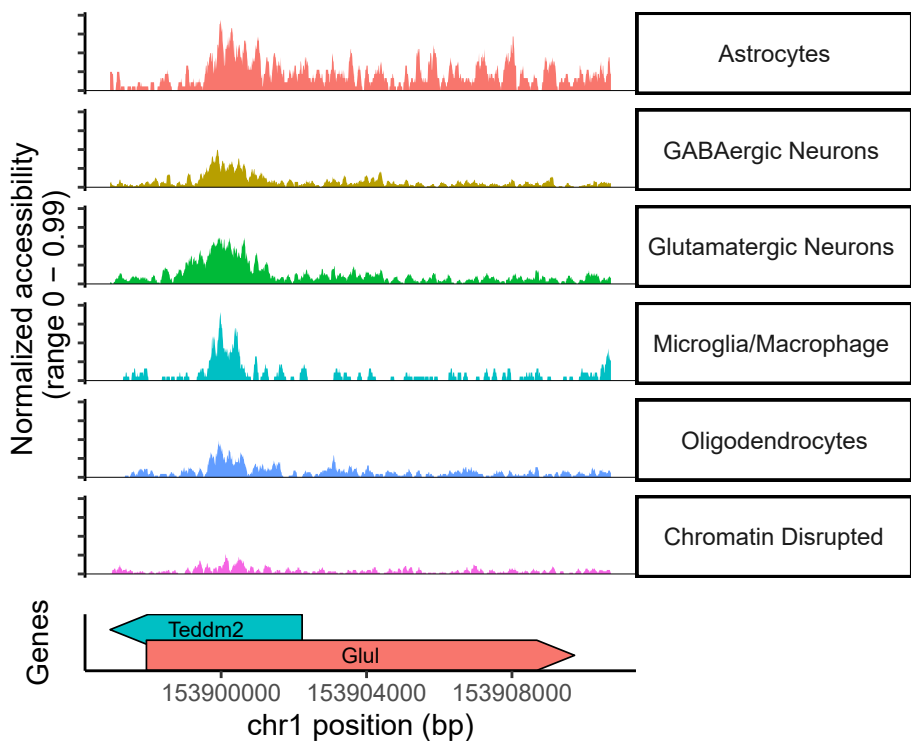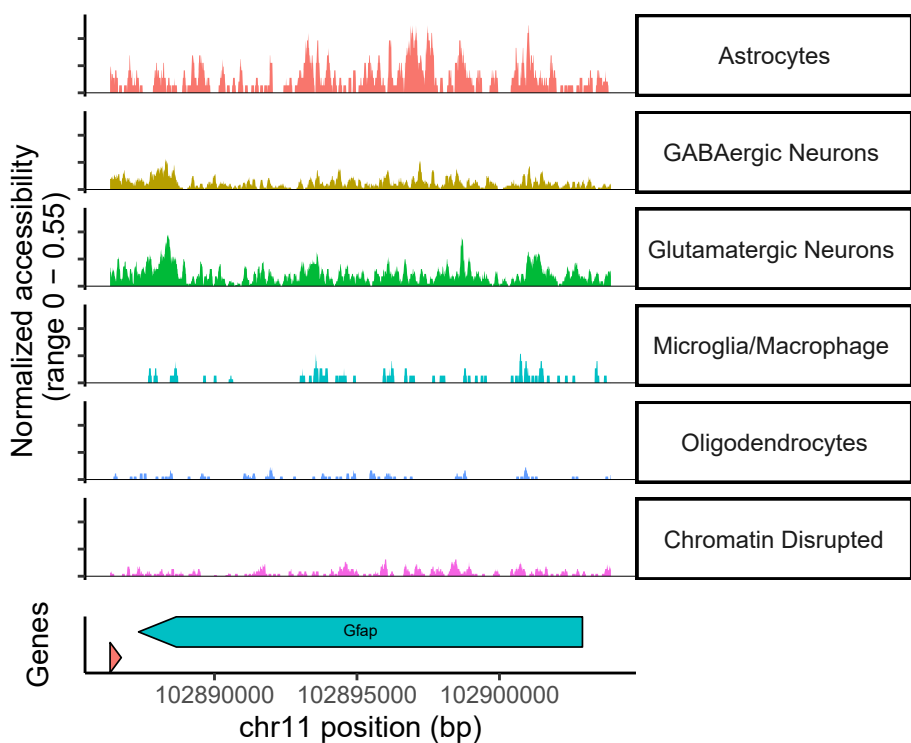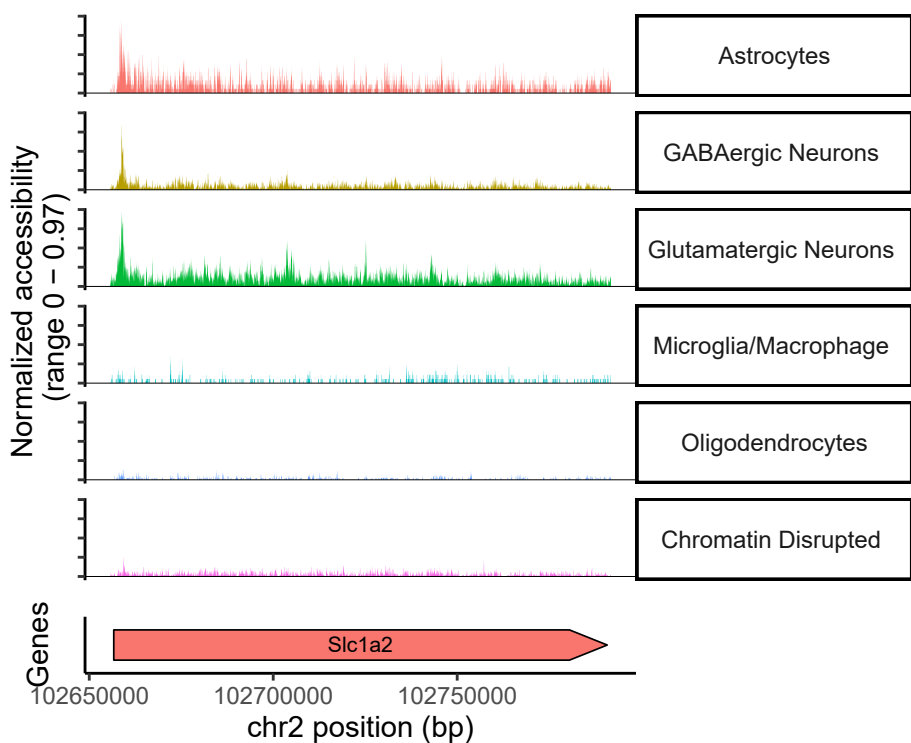

Supplement: Supplementary file 3 — Supplementary Data 1 [file 41467_2021_21515_MOESM3_ESM.zip › Corrected_CelltypeMarker_Plots/MouseCerebralIschemia.markeraccessibility/MouseCerebralIschemia.Astro.markeraccessibility.pdf]

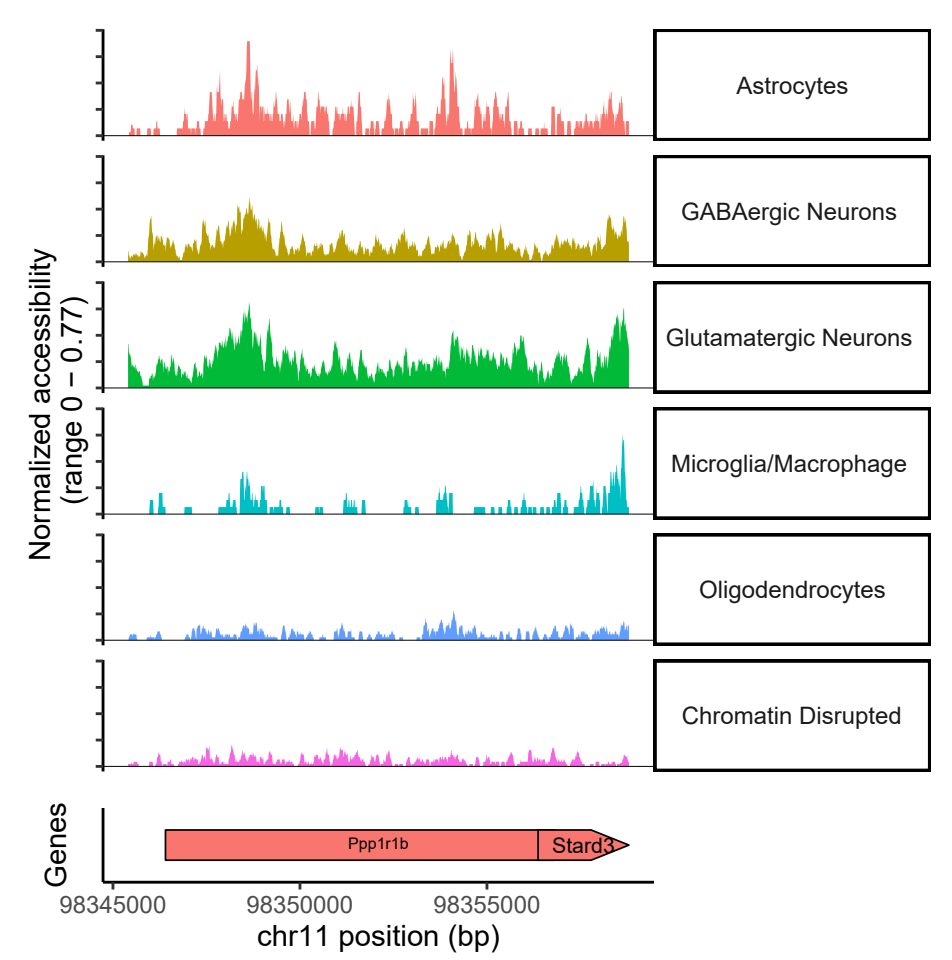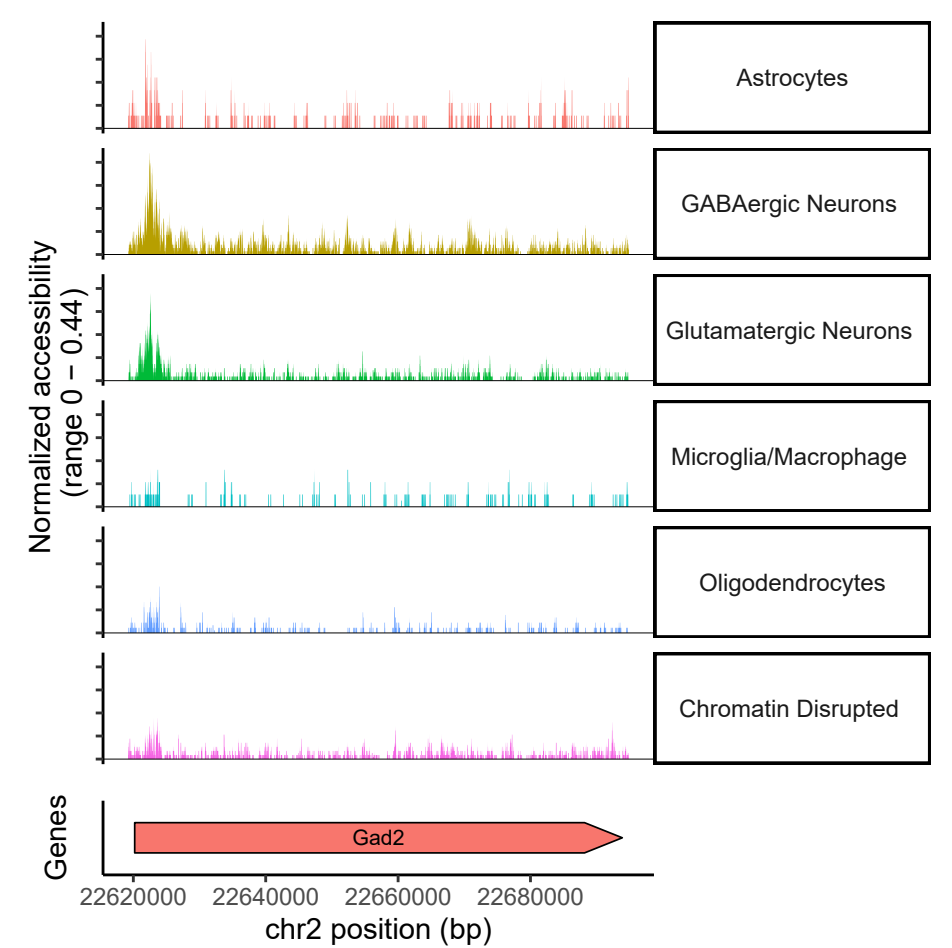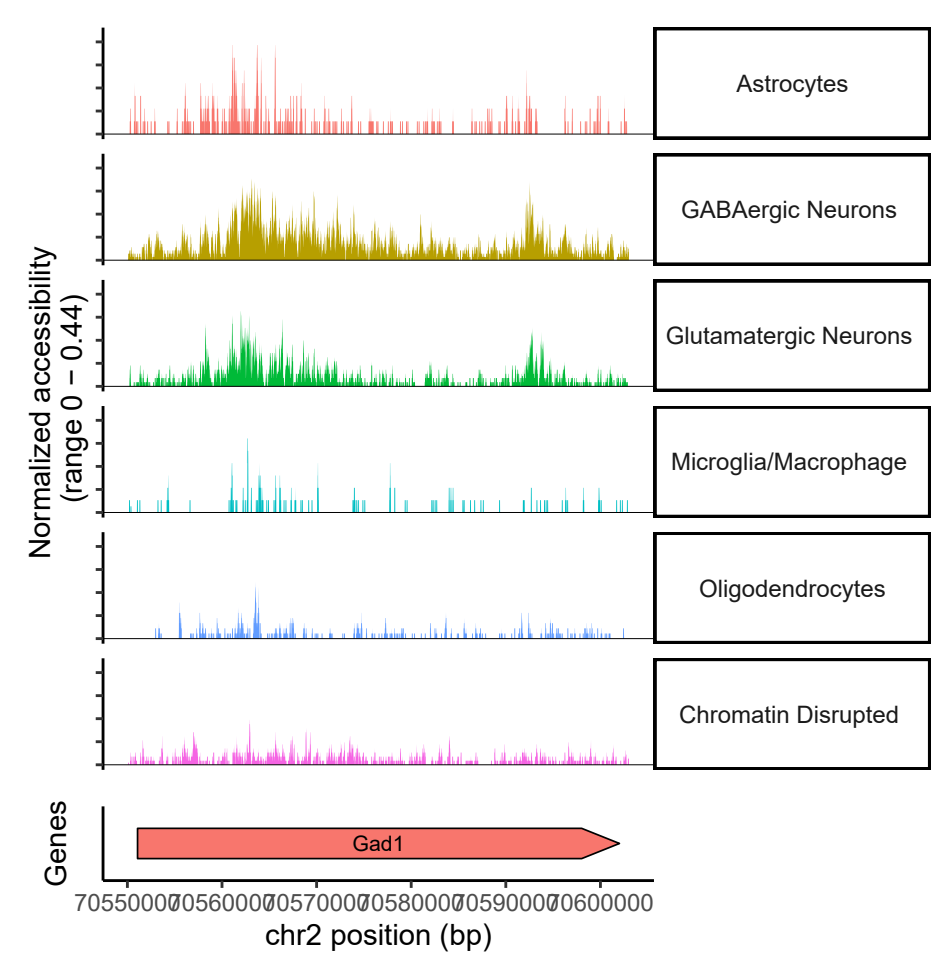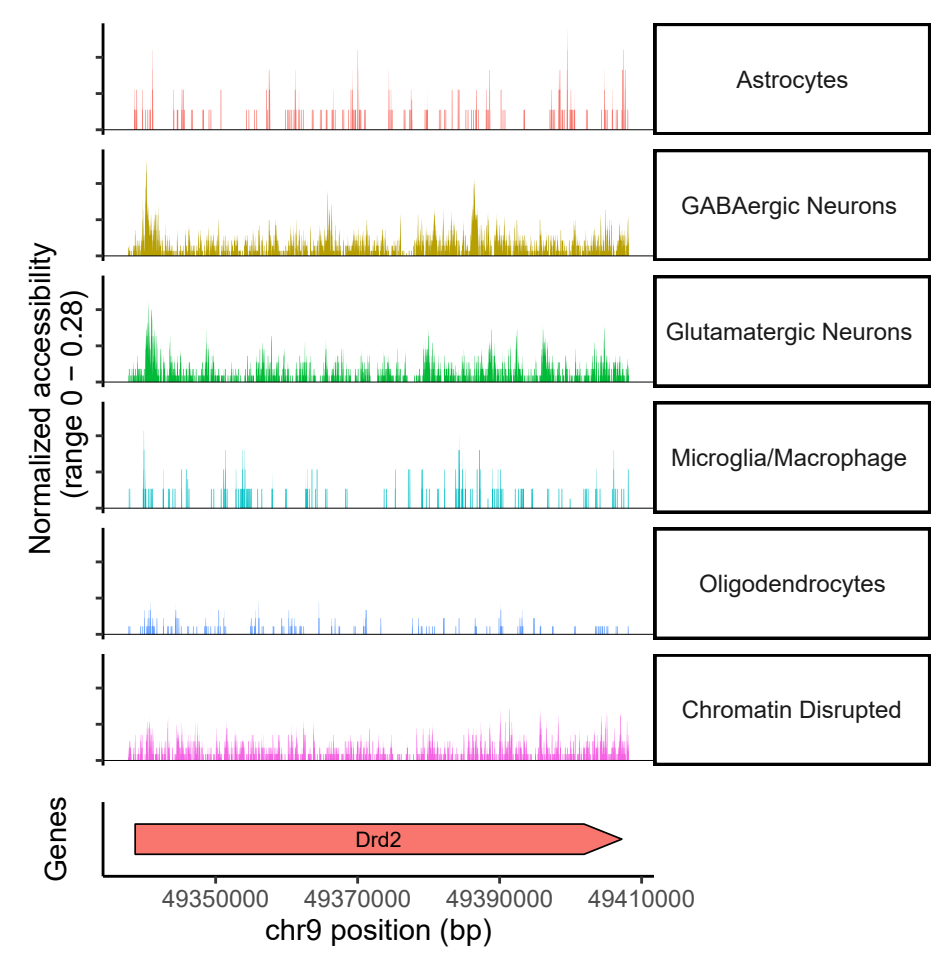

Supplement: Supplementary file 3 — Supplementary Data 1 [file 41467_2021_21515_MOESM3_ESM.zip › Corrected_CelltypeMarker_Plots/MouseCerebralIschemia.markeraccessibility/MouseCerebralIschemia.GABA.markeraccessibility.pdf]

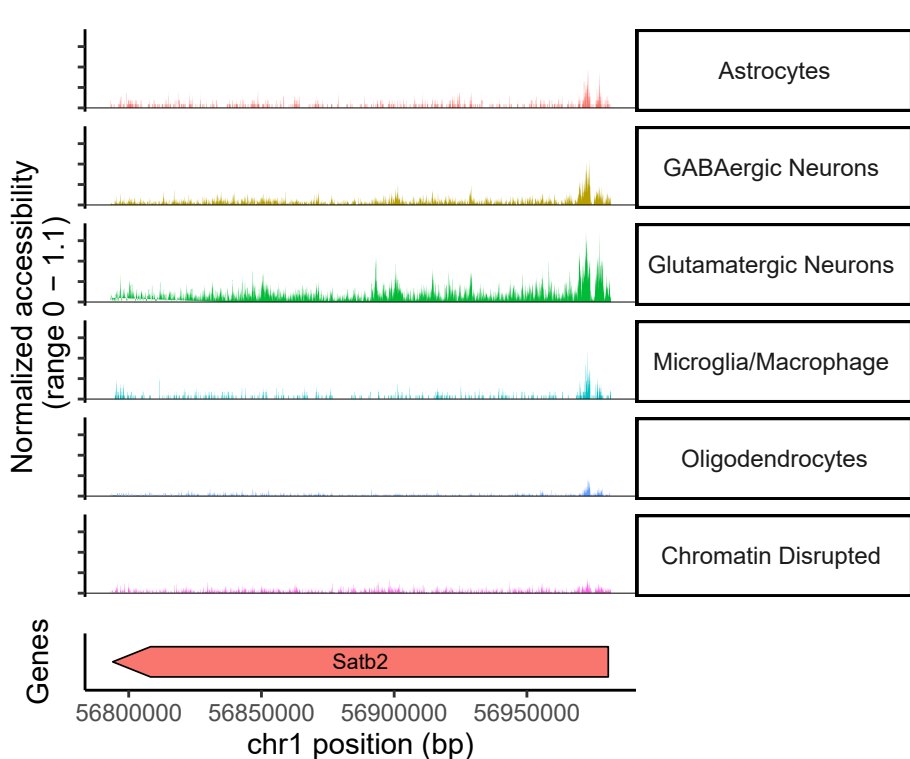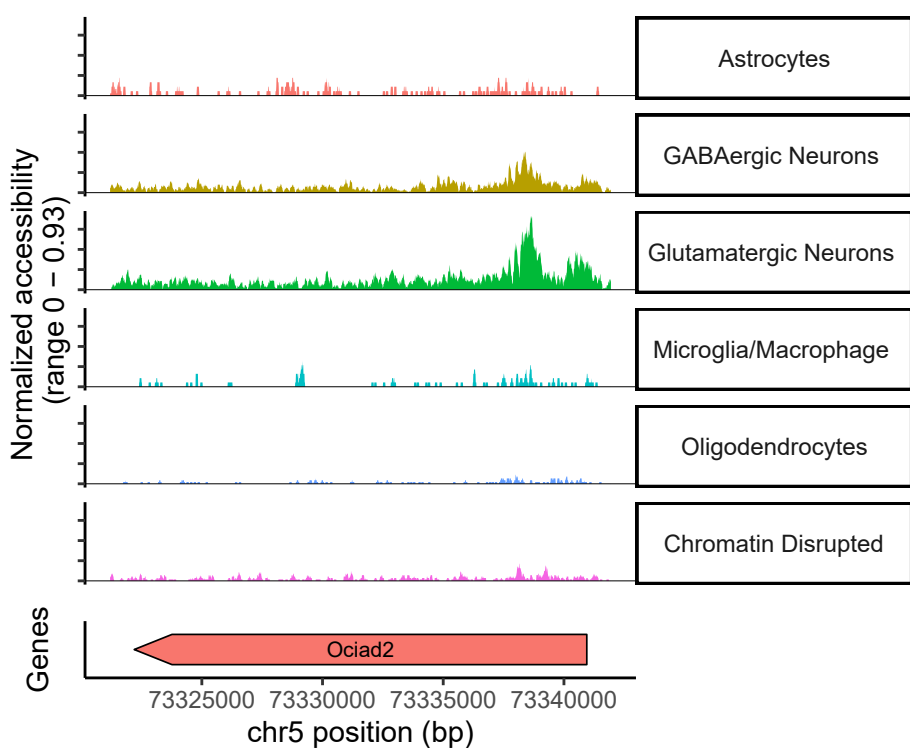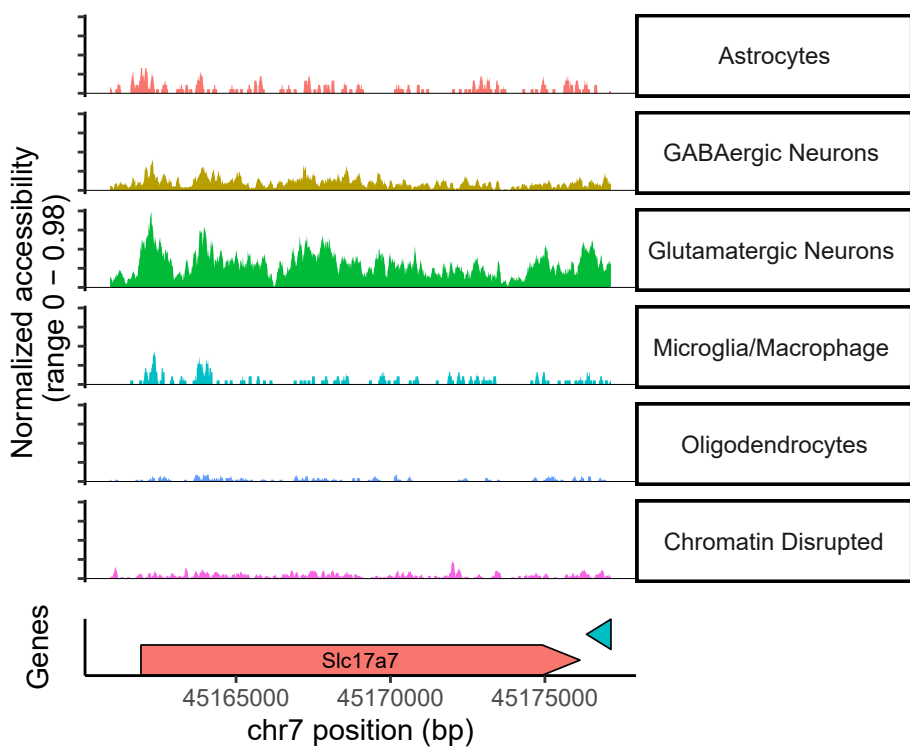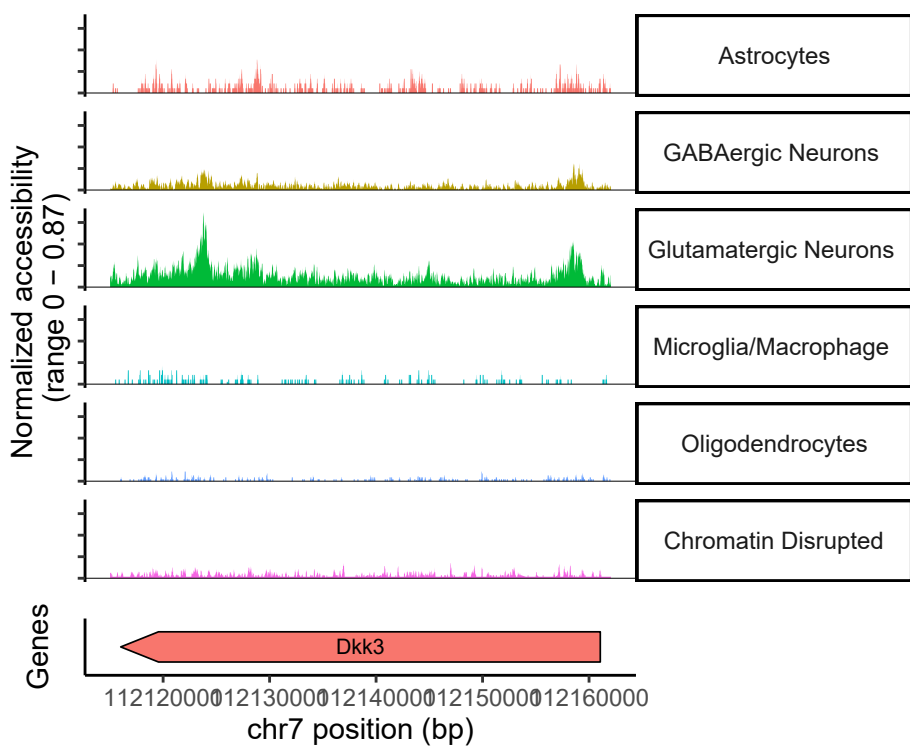

Supplement: Supplementary file 3 — Supplementary Data 1 [file 41467_2021_21515_MOESM3_ESM.zip › Corrected_CelltypeMarker_Plots/MouseCerebralIschemia.markeraccessibility/MouseCerebralIschemia.Glut.markeraccessibility.pdf]

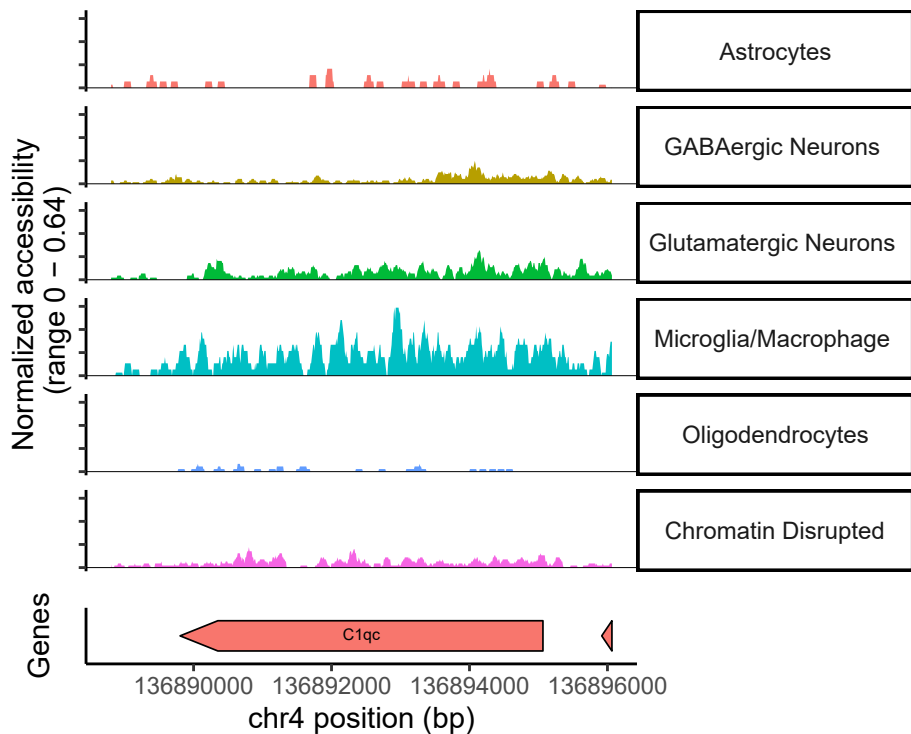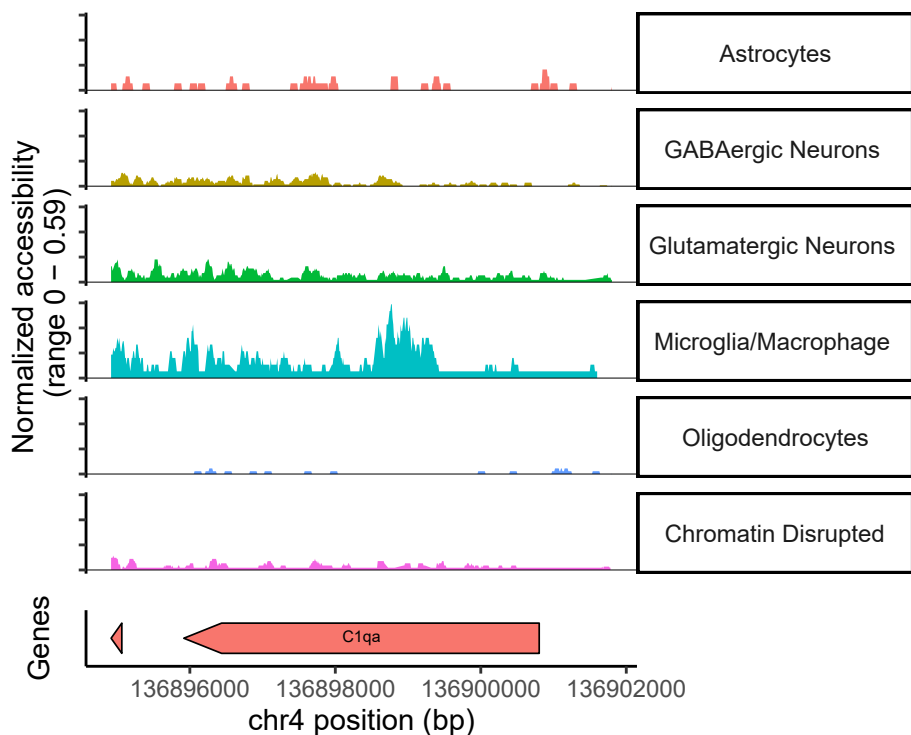

Supplement: Supplementary file 3 — Supplementary Data 1 [file 41467_2021_21515_MOESM3_ESM.zip › Corrected_CelltypeMarker_Plots/MouseCerebralIschemia.markeraccessibility/MouseCerebralIschemia.Micro.markeraccessibility.pdf]

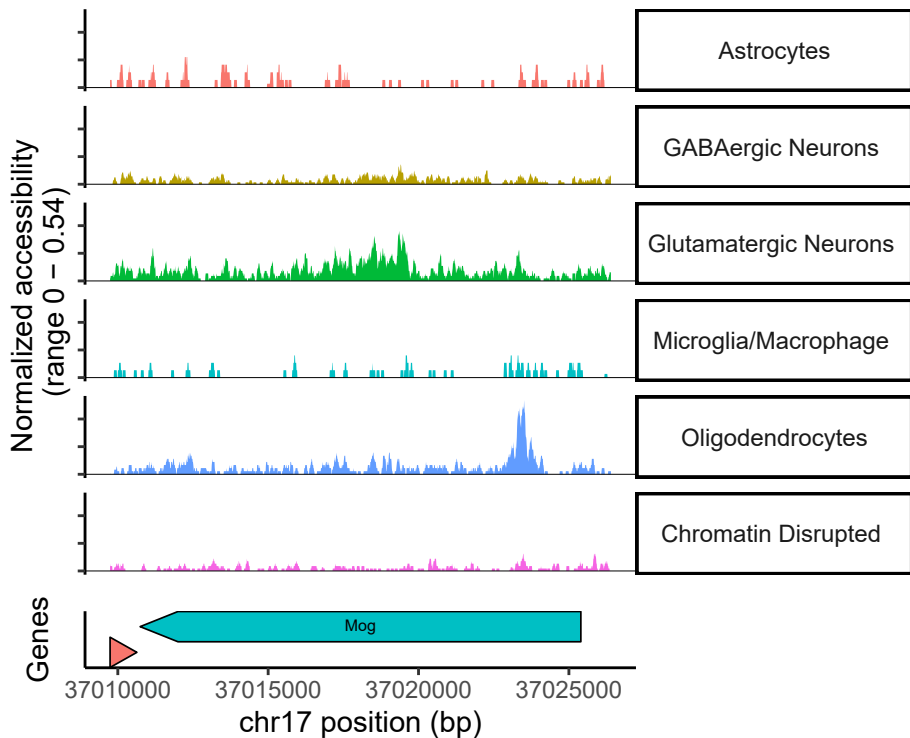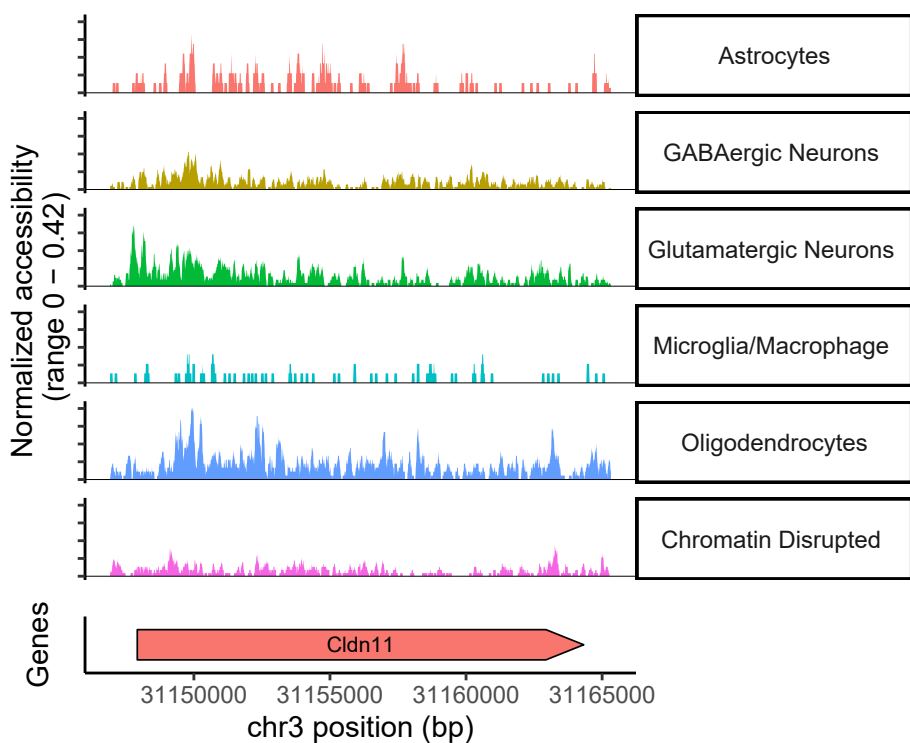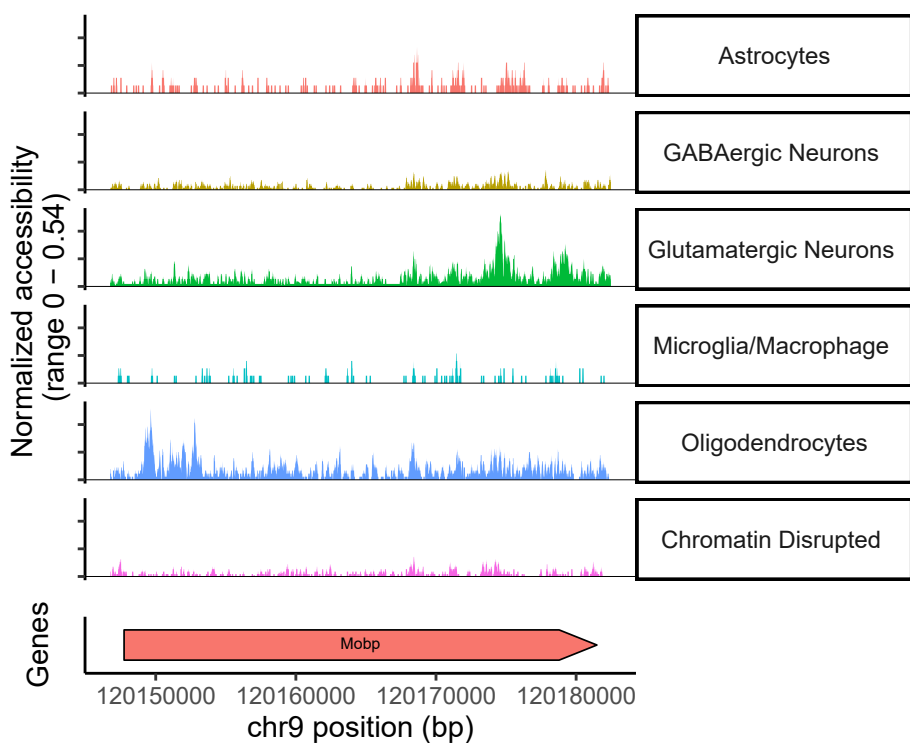

Supplement: Supplementary file 3 — Supplementary Data 1 [file 41467_2021_21515_MOESM3_ESM.zip › Corrected_CelltypeMarker_Plots/MouseCerebralIschemia.markeraccessibility/MouseCerebralIschemia.Olig.markeraccessibility.pdf]

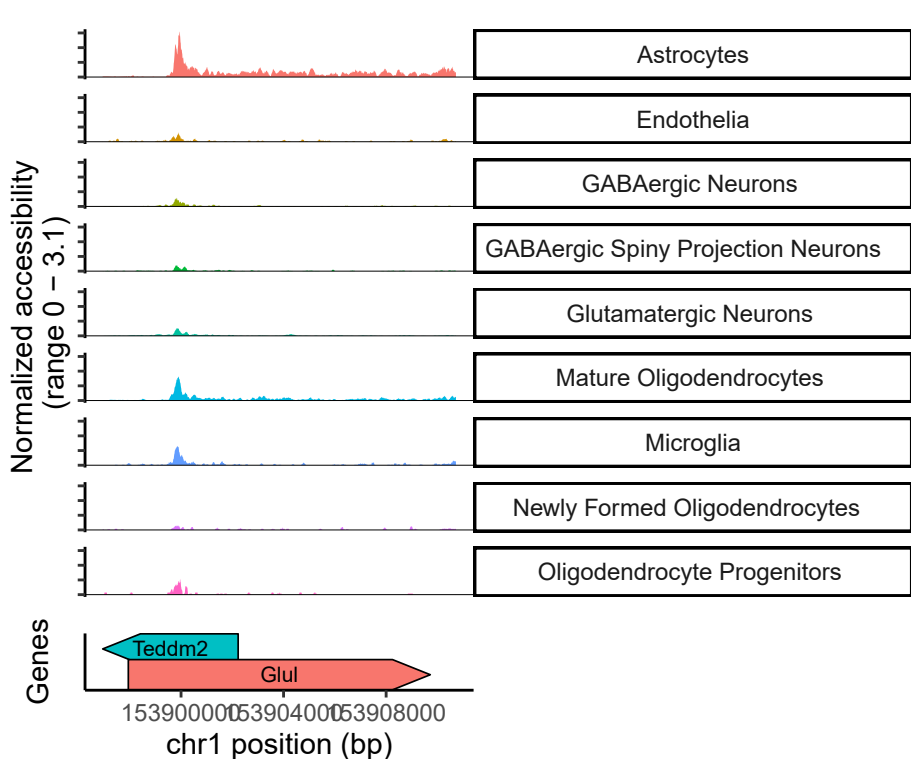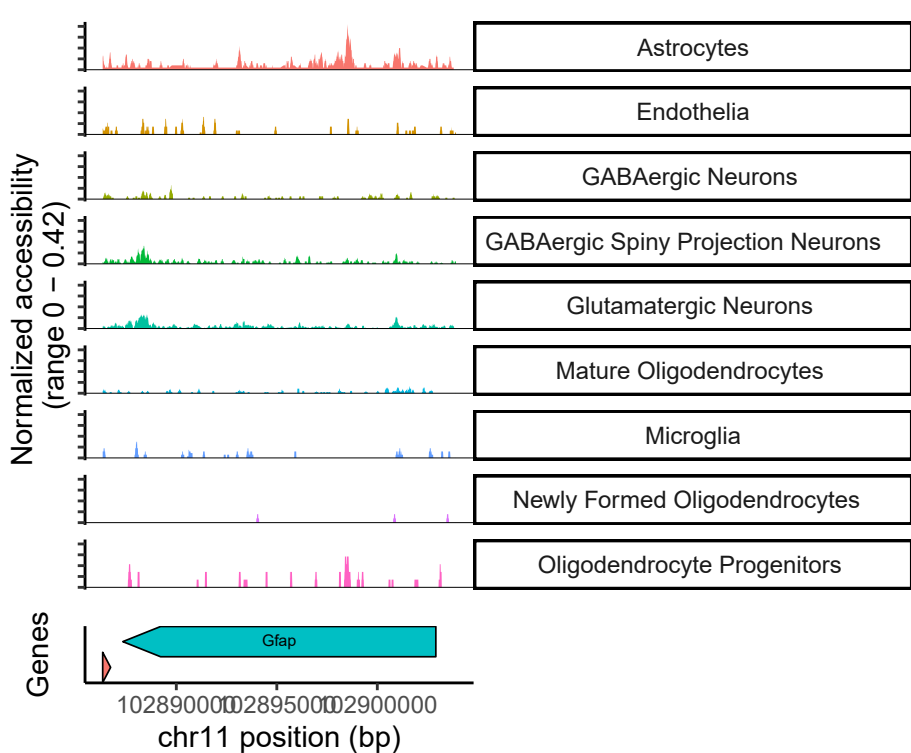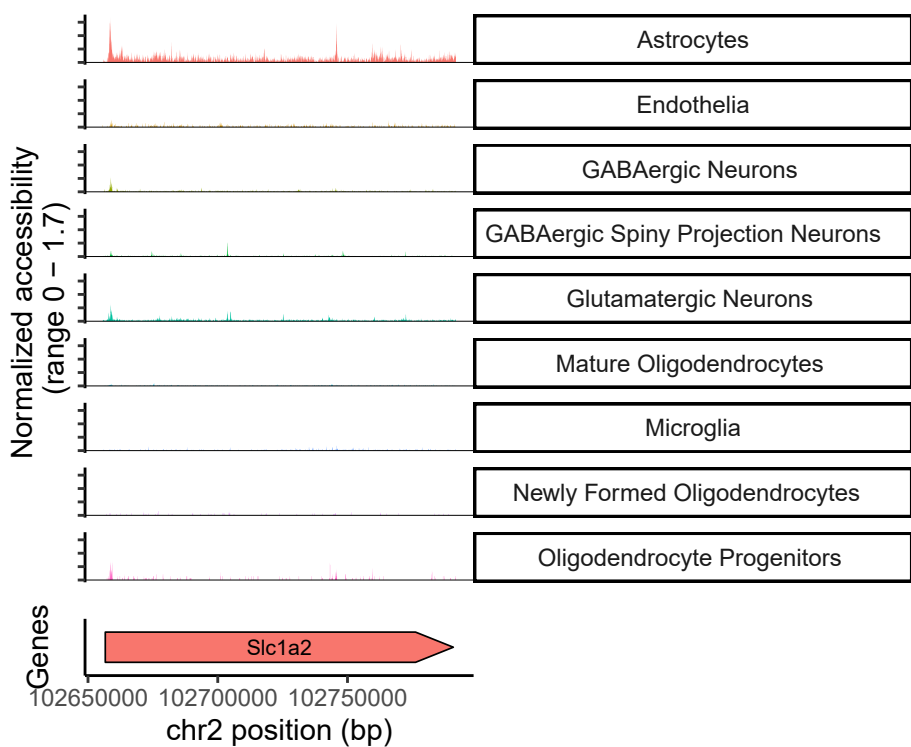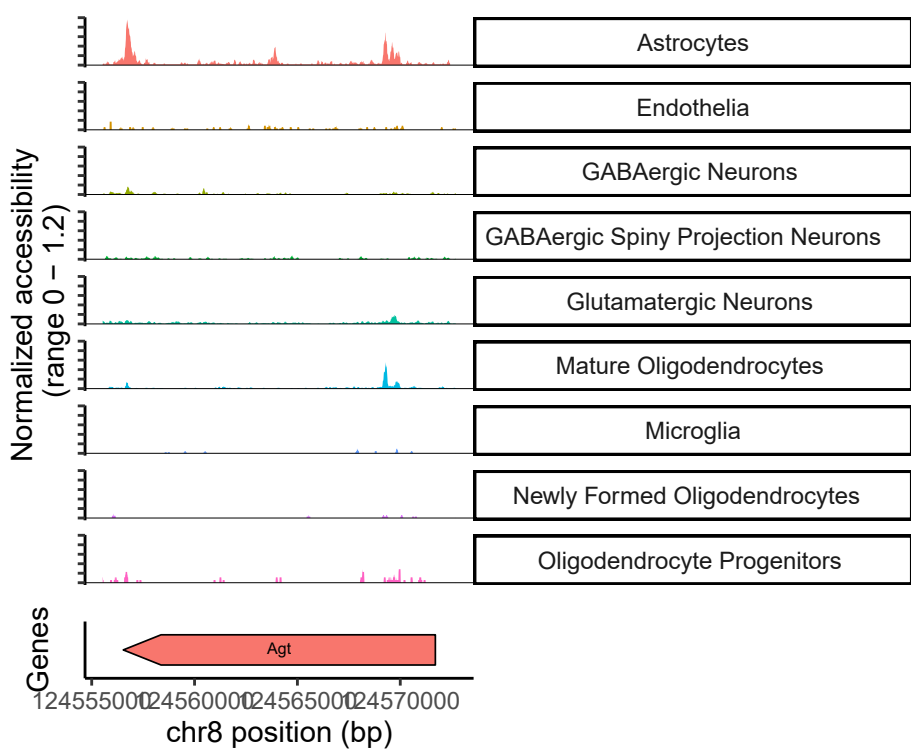

Supplement: Supplementary file 3 — Supplementary Data 1 [file 41467_2021_21515_MOESM3_ESM.zip › Corrected_CelltypeMarker_Plots/MouseSSpCortex.markeraccessibility/MouseSSpCortex.Astro.markeraccessibility.pdf]

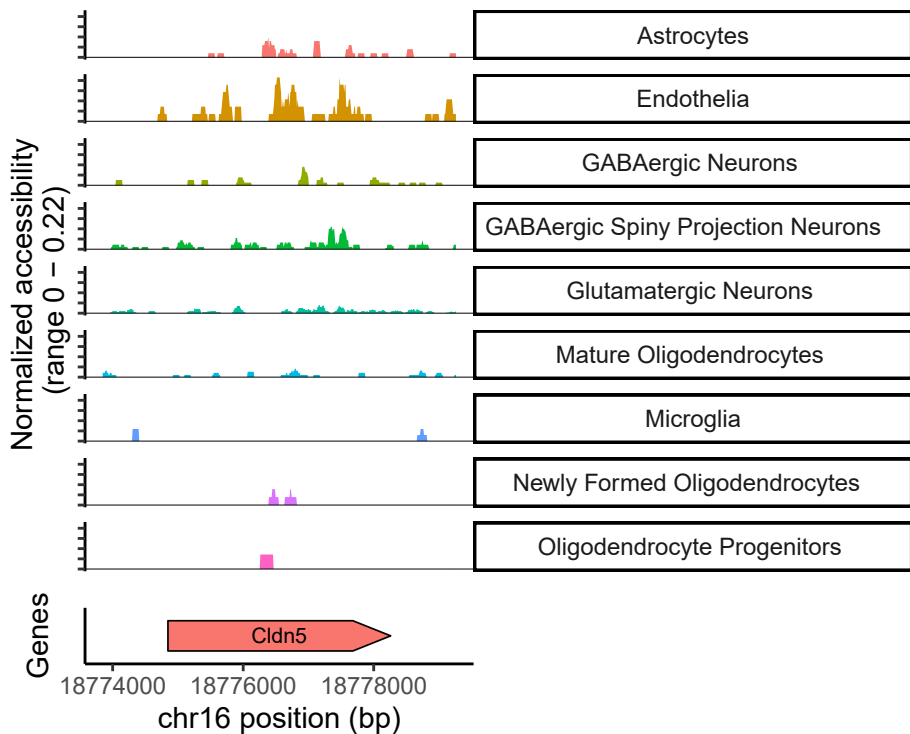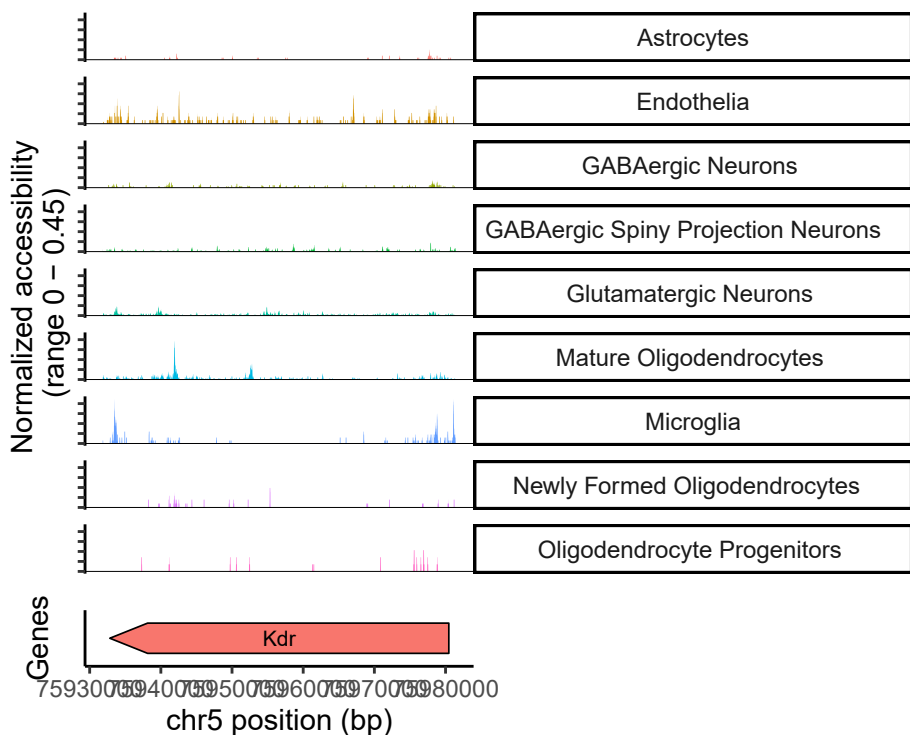

Supplement: Supplementary file 3 — Supplementary Data 1 [file 41467_2021_21515_MOESM3_ESM.zip › Corrected_CelltypeMarker_Plots/MouseSSpCortex.markeraccessibility/MouseSSpCortex.Endo.markeraccessibility.pdf]

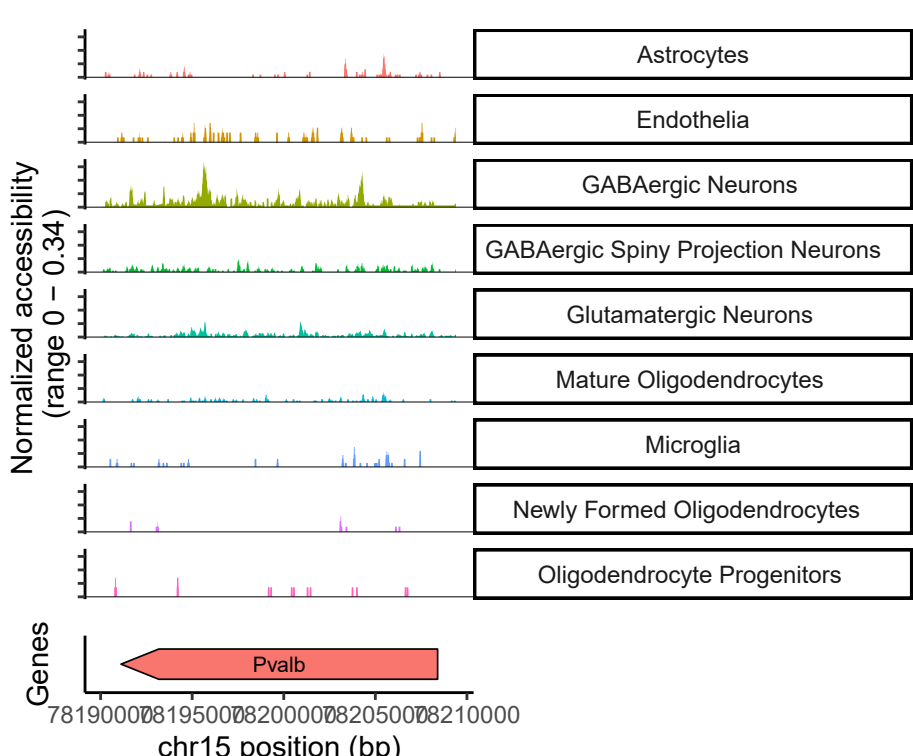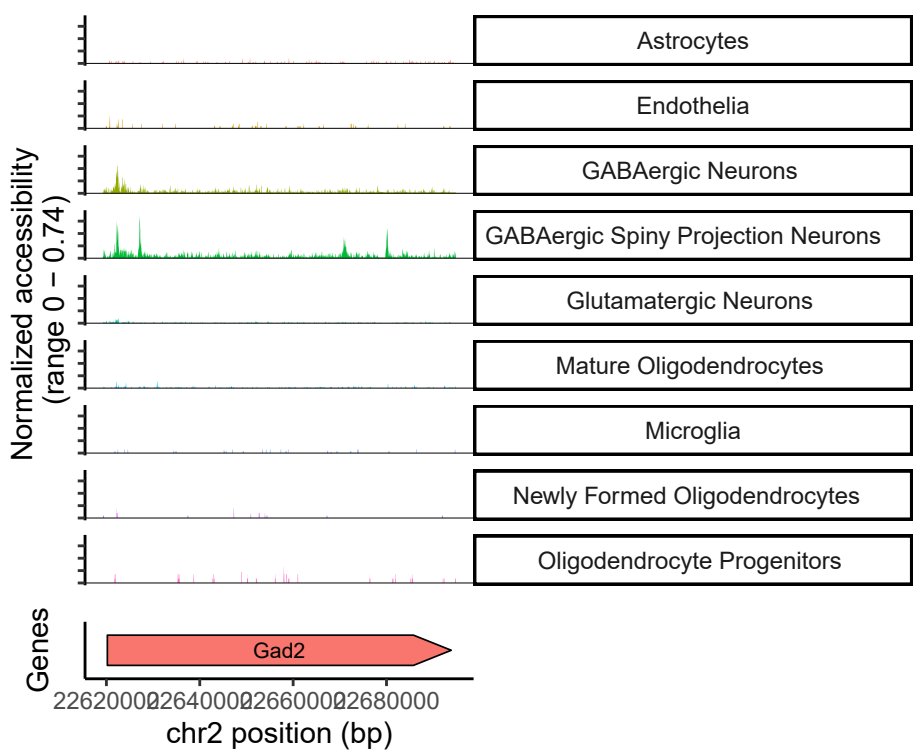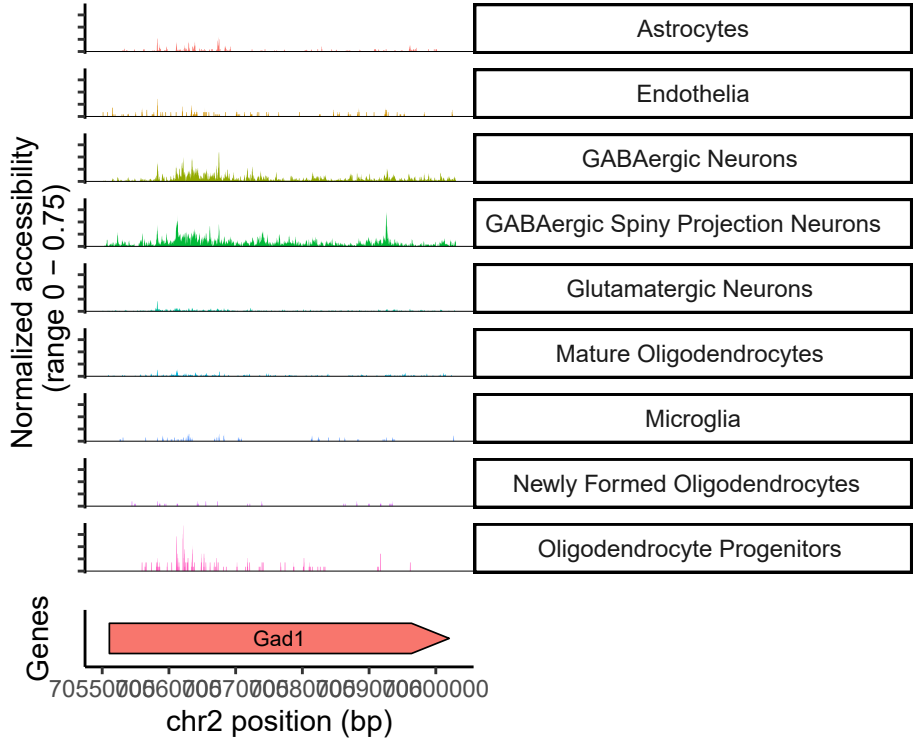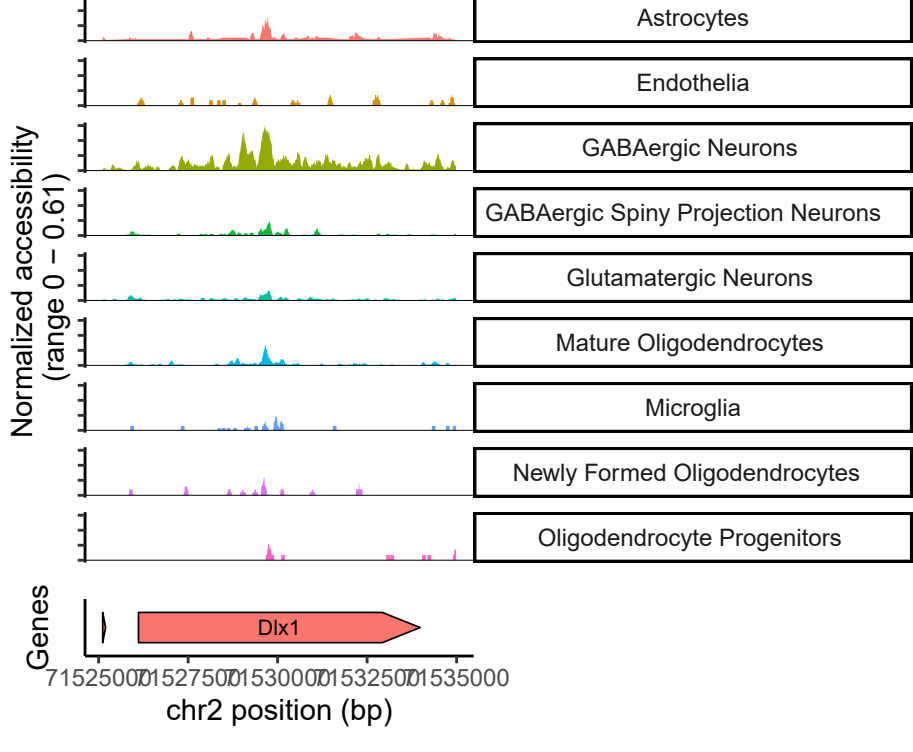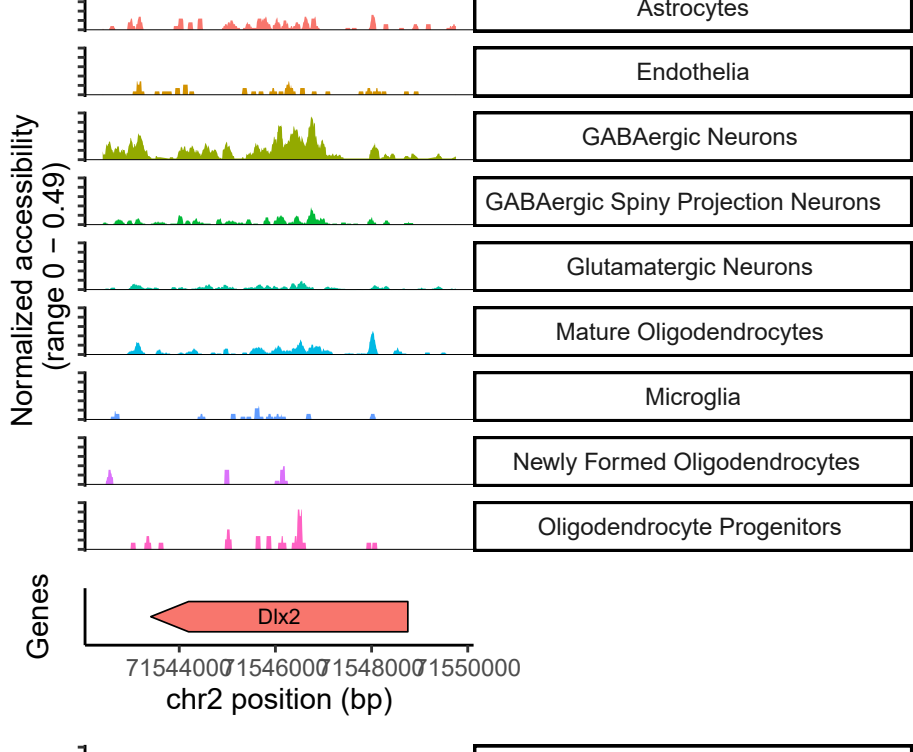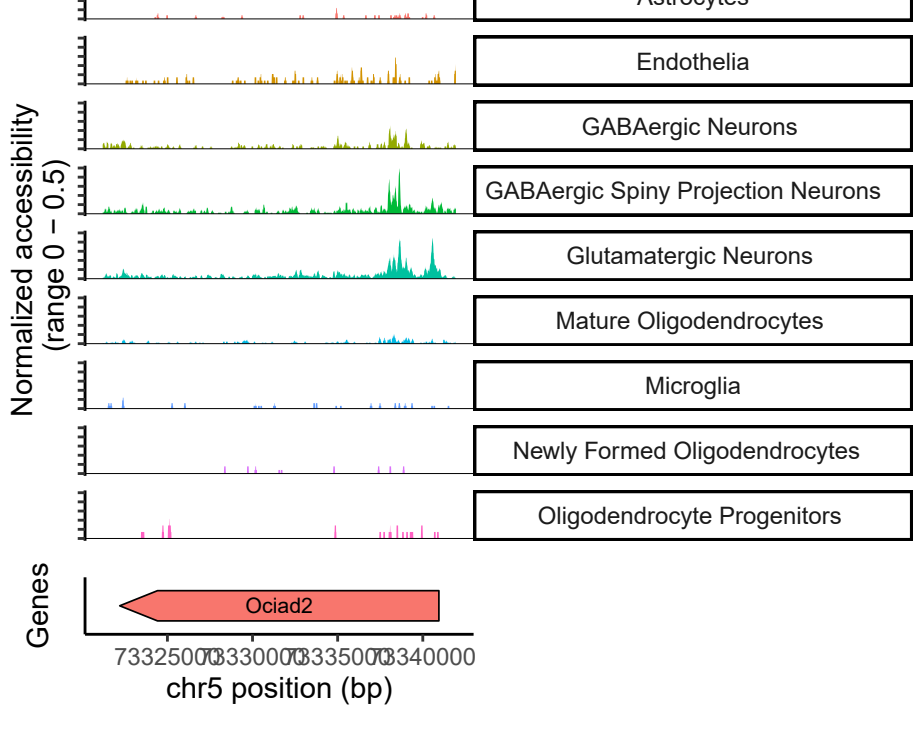

Supplement: Supplementary file 3 — Supplementary Data 1 [file 41467_2021_21515_MOESM3_ESM.zip › Corrected_CelltypeMarker_Plots/MouseSSpCortex.markeraccessibility/MouseSSpCortex.GABA.markeraccessibility.pdf]

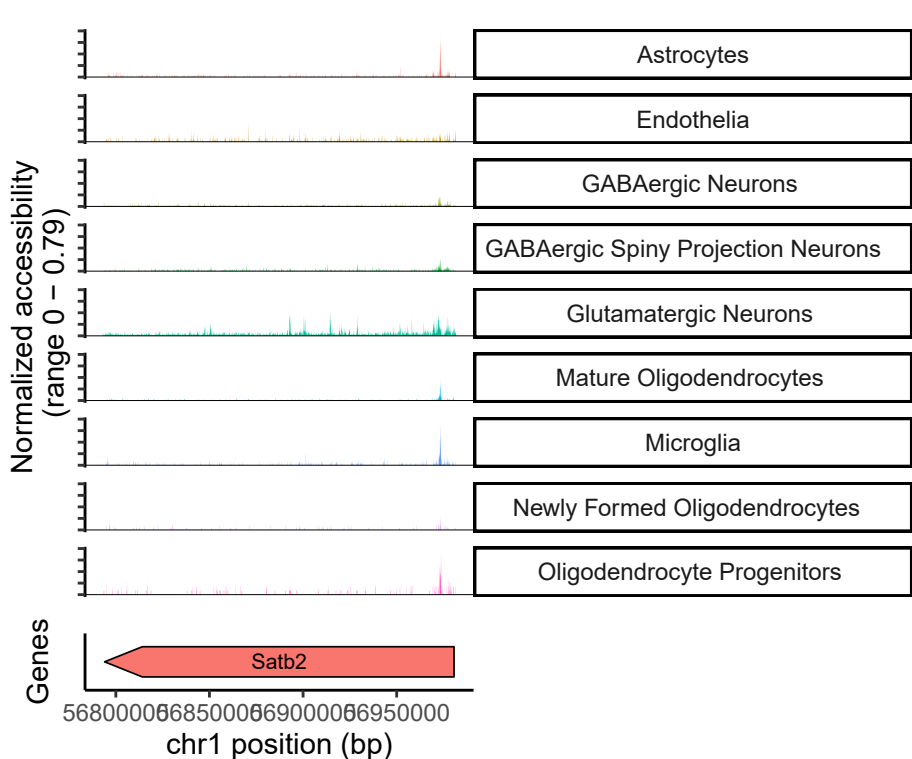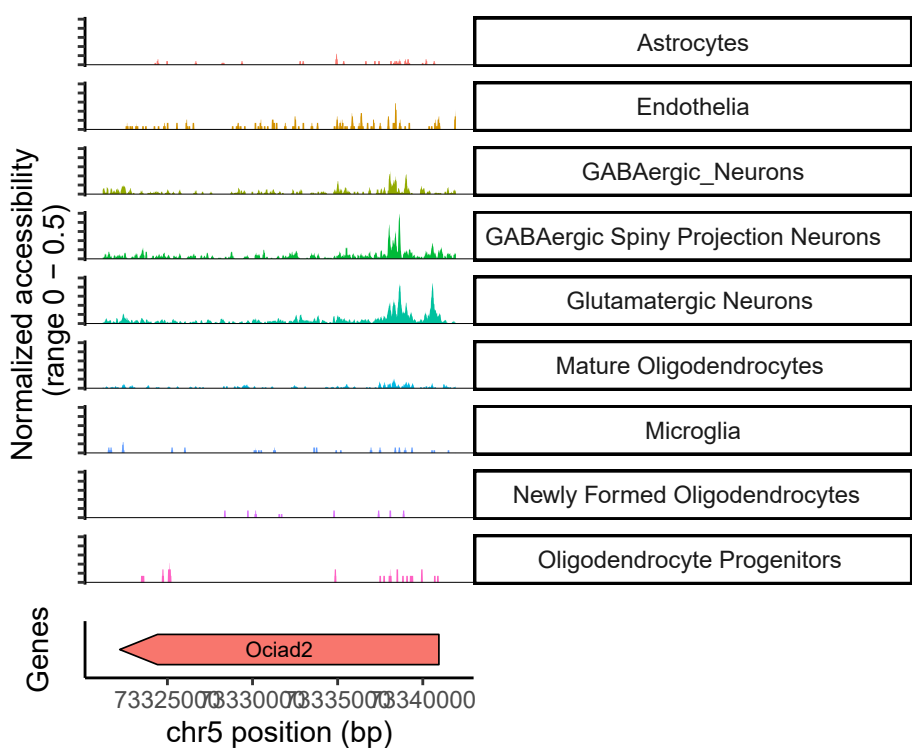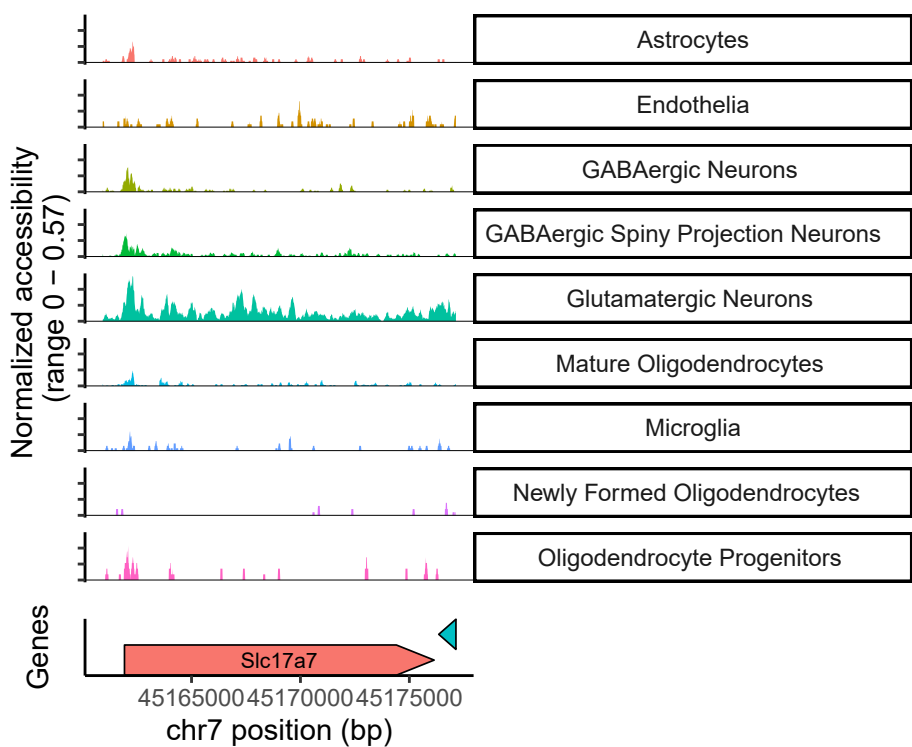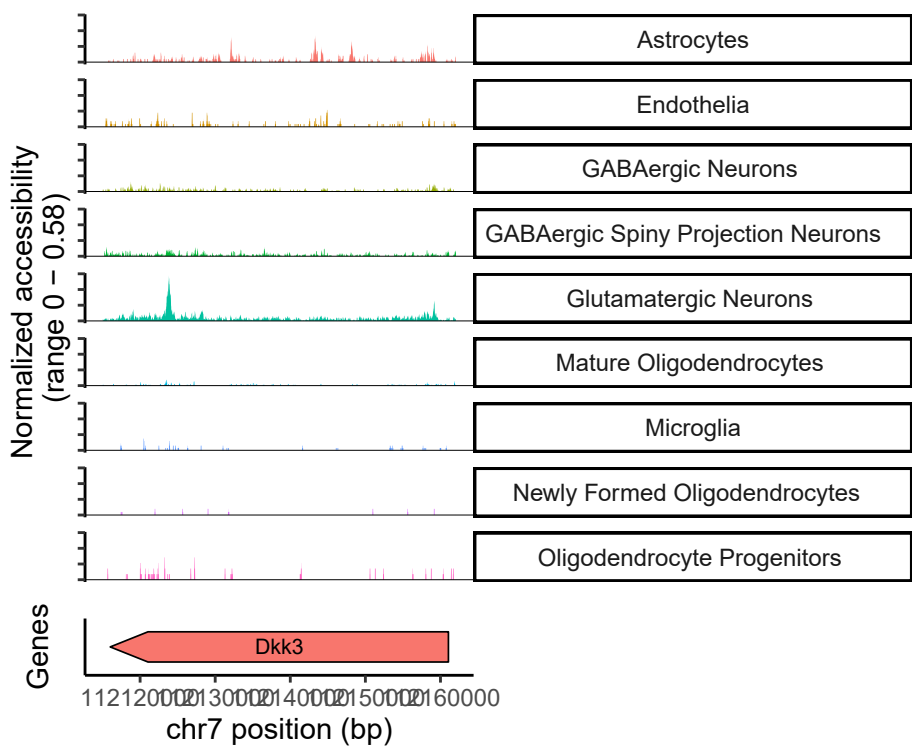

Supplement: Supplementary file 3 — Supplementary Data 1 [file 41467_2021_21515_MOESM3_ESM.zip › Corrected_CelltypeMarker_Plots/MouseSSpCortex.markeraccessibility/MouseSSpCortex.Glut.markeraccessibility.pdf]

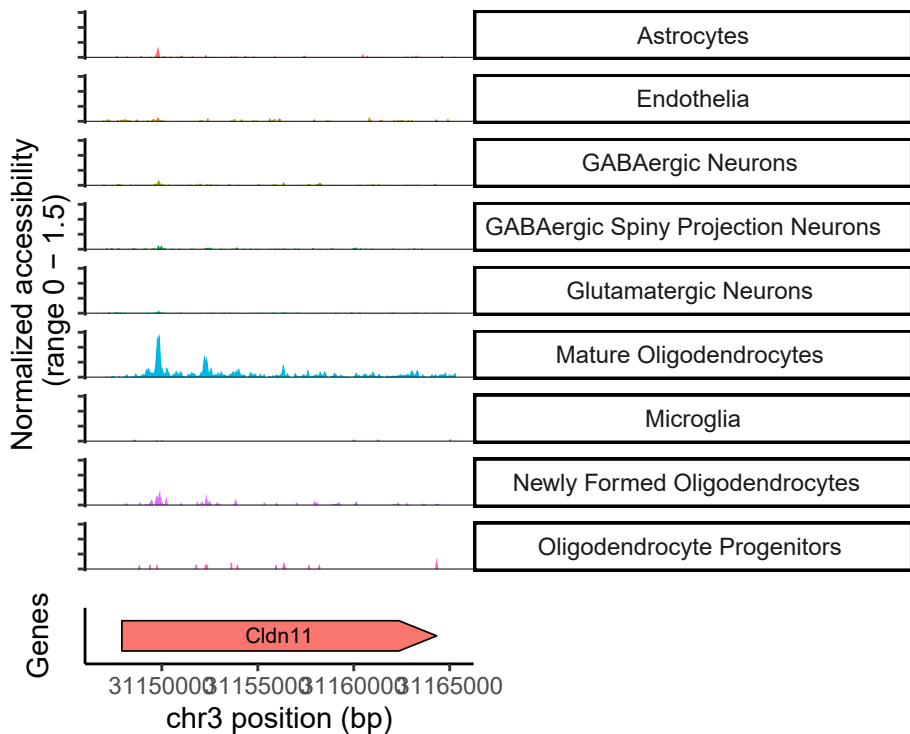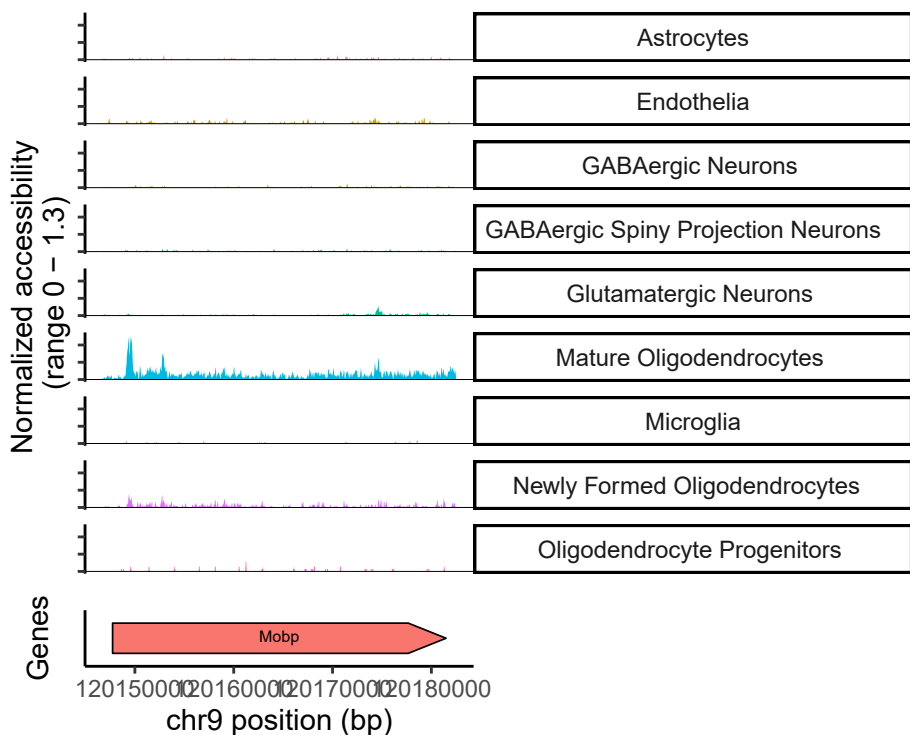

Supplement: Supplementary file 3 — Supplementary Data 1 [file 41467_2021_21515_MOESM3_ESM.zip › Corrected_CelltypeMarker_Plots/MouseSSpCortex.markeraccessibility/MouseSSpCortex.IntOligo.markeraccessibility.pdf]

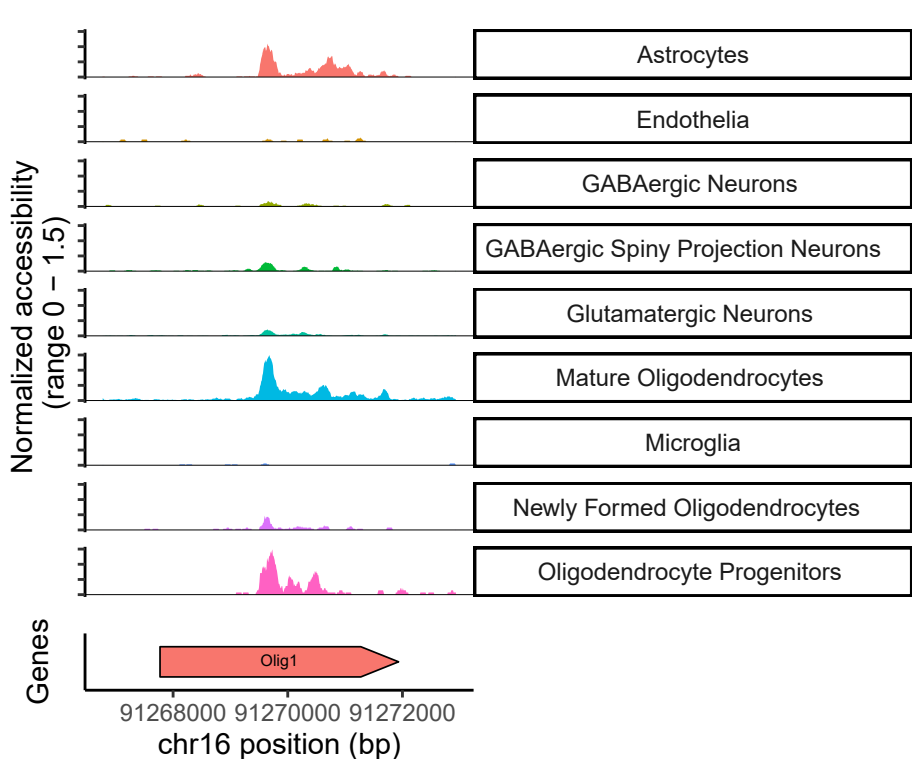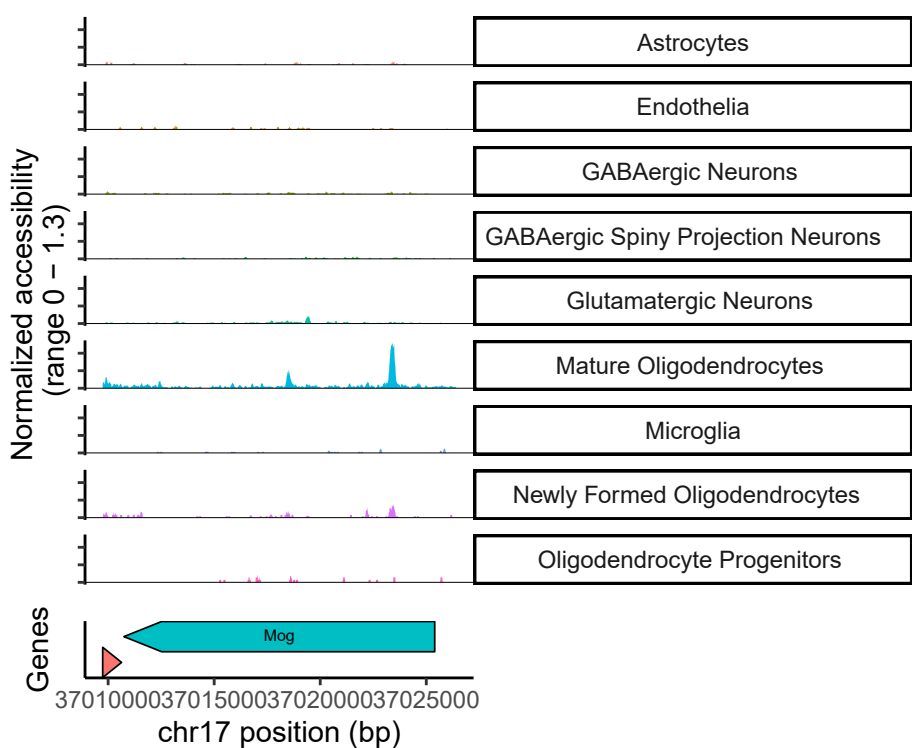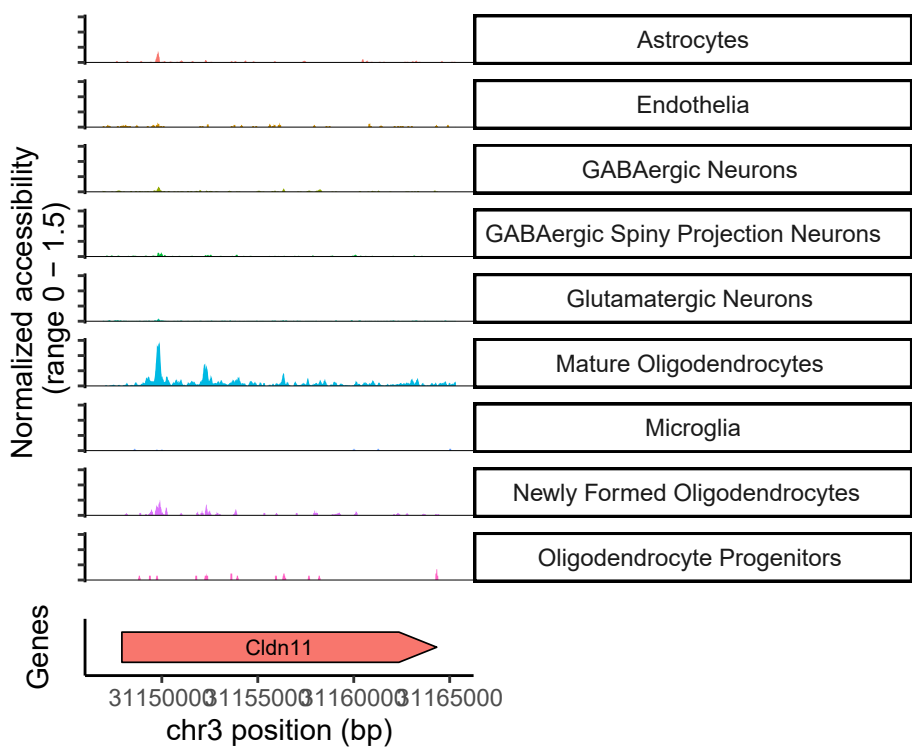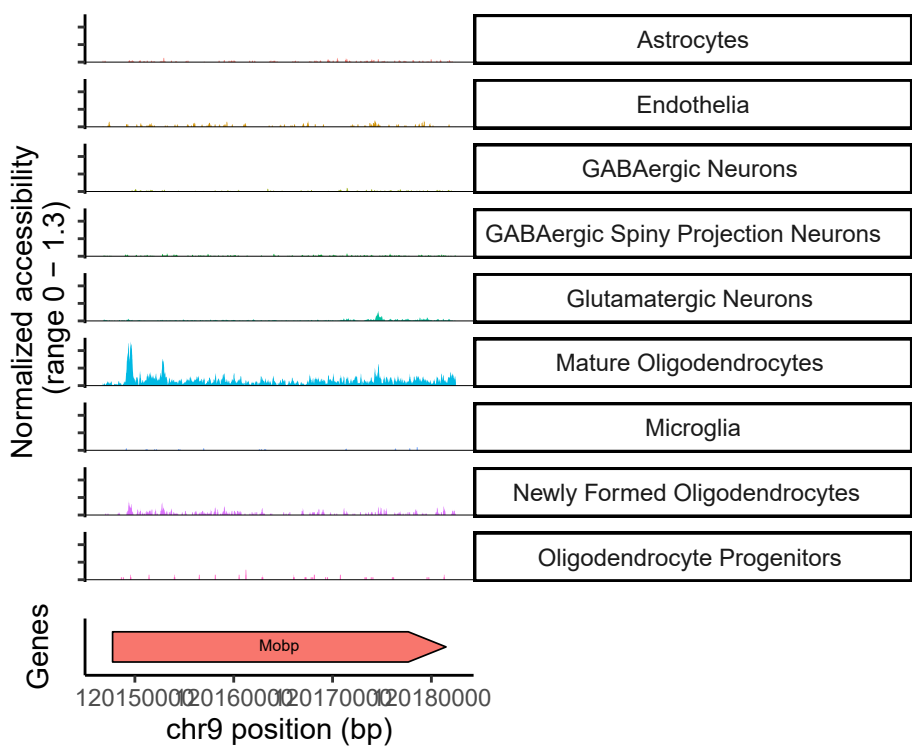

Supplement: Supplementary file 3 — Supplementary Data 1 [file 41467_2021_21515_MOESM3_ESM.zip › Corrected_CelltypeMarker_Plots/MouseSSpCortex.markeraccessibility/MouseSSpCortex.MatOligo.markeraccessibility.pdf]

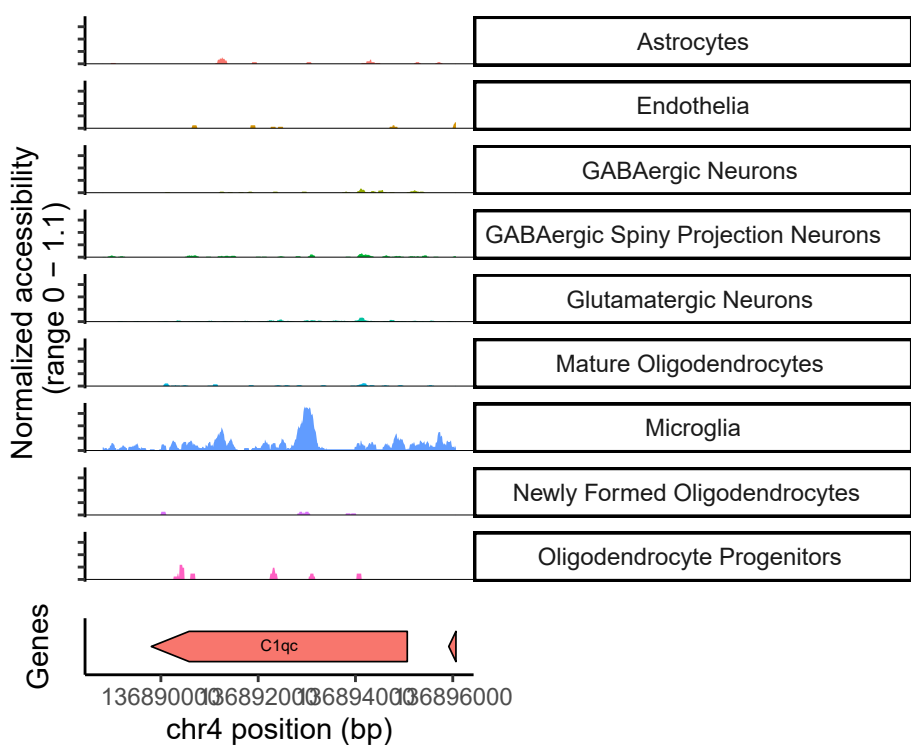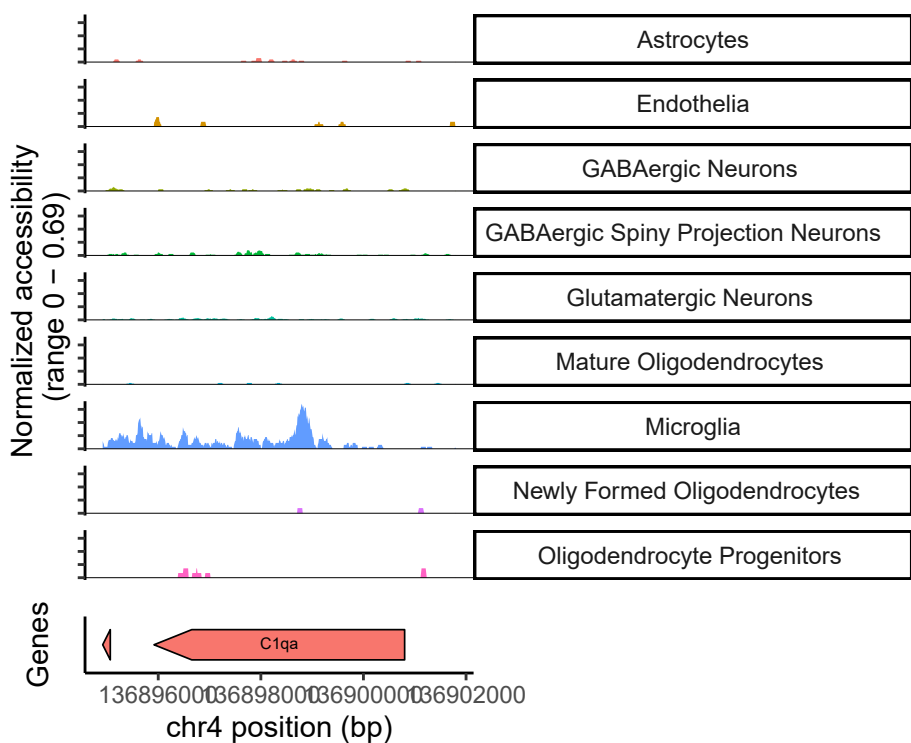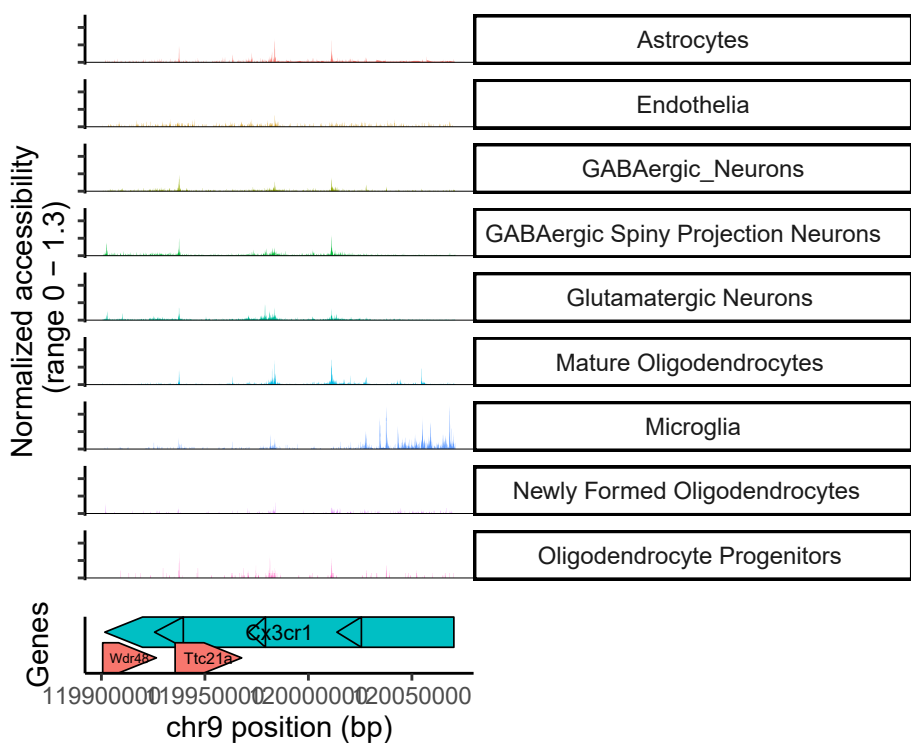

Supplement: Supplementary file 3 — Supplementary Data 1 [file 41467_2021_21515_MOESM3_ESM.zip › Corrected_CelltypeMarker_Plots/MouseSSpCortex.markeraccessibility/MouseSSpCortex.Micro.markeraccessibility.pdf]

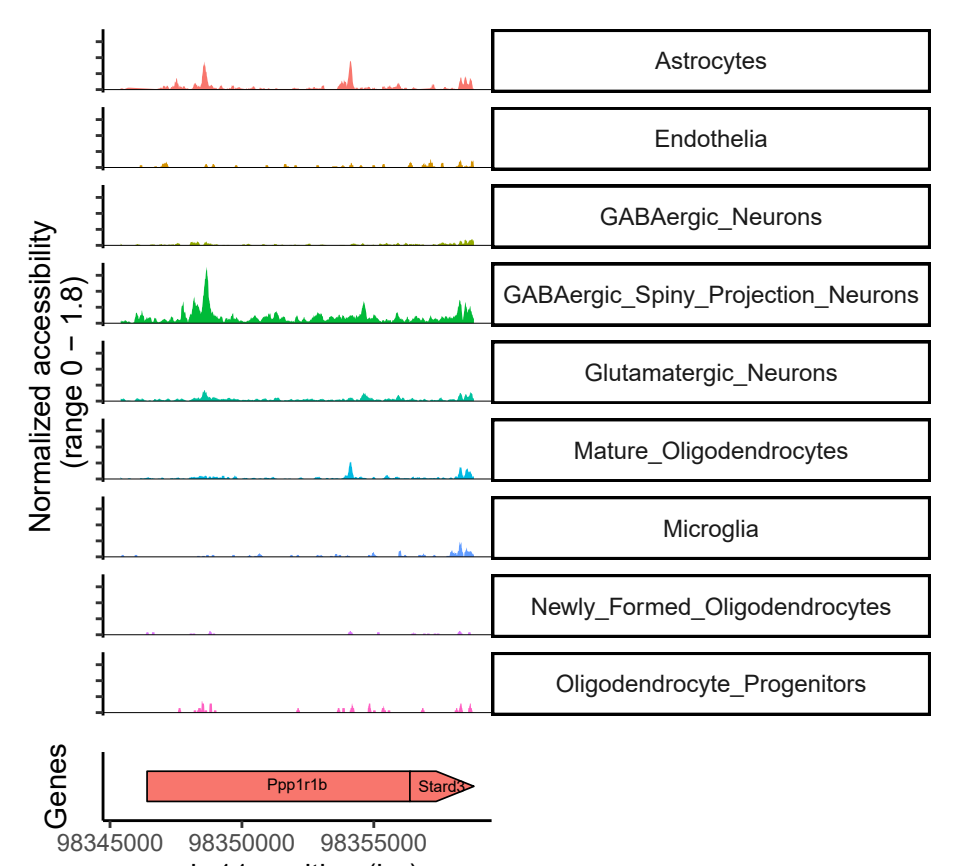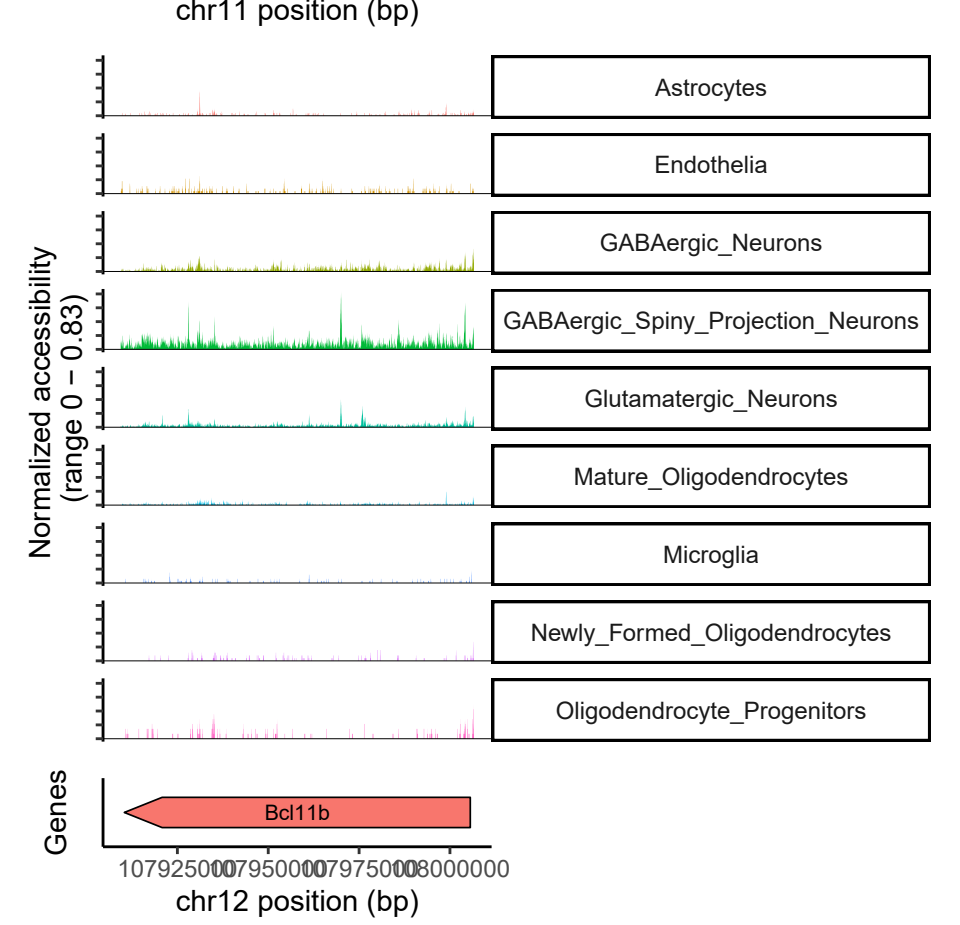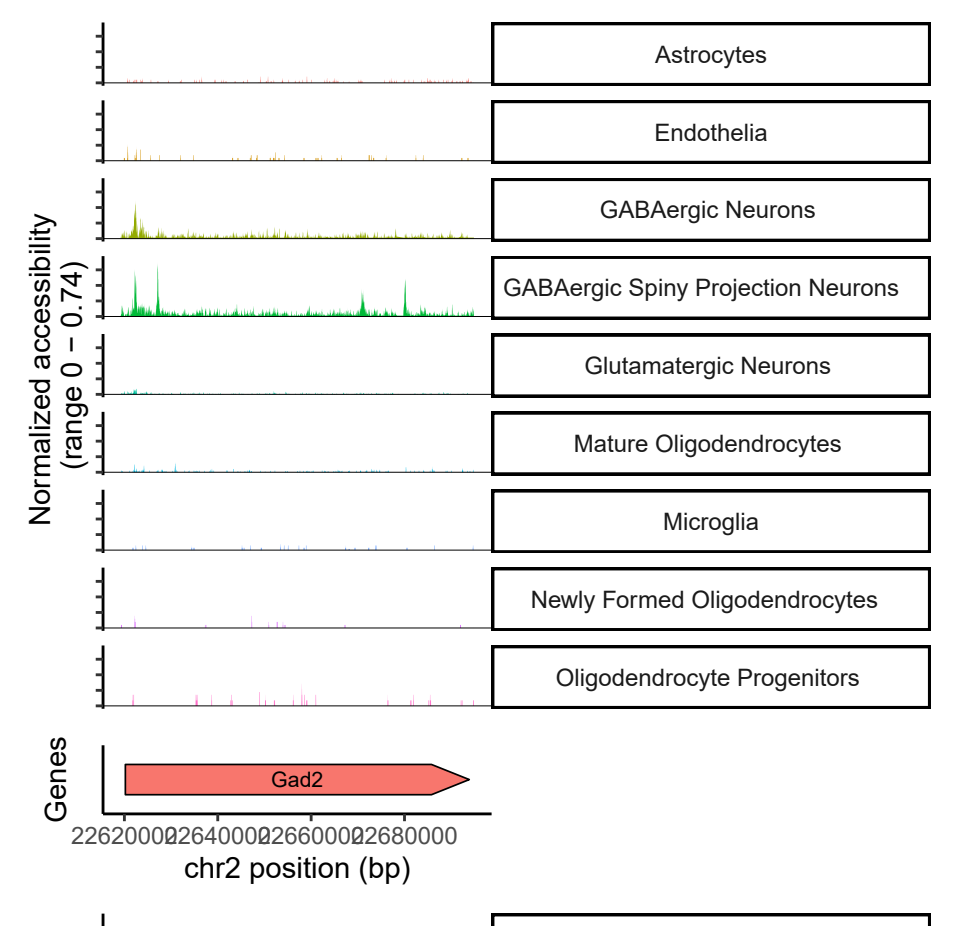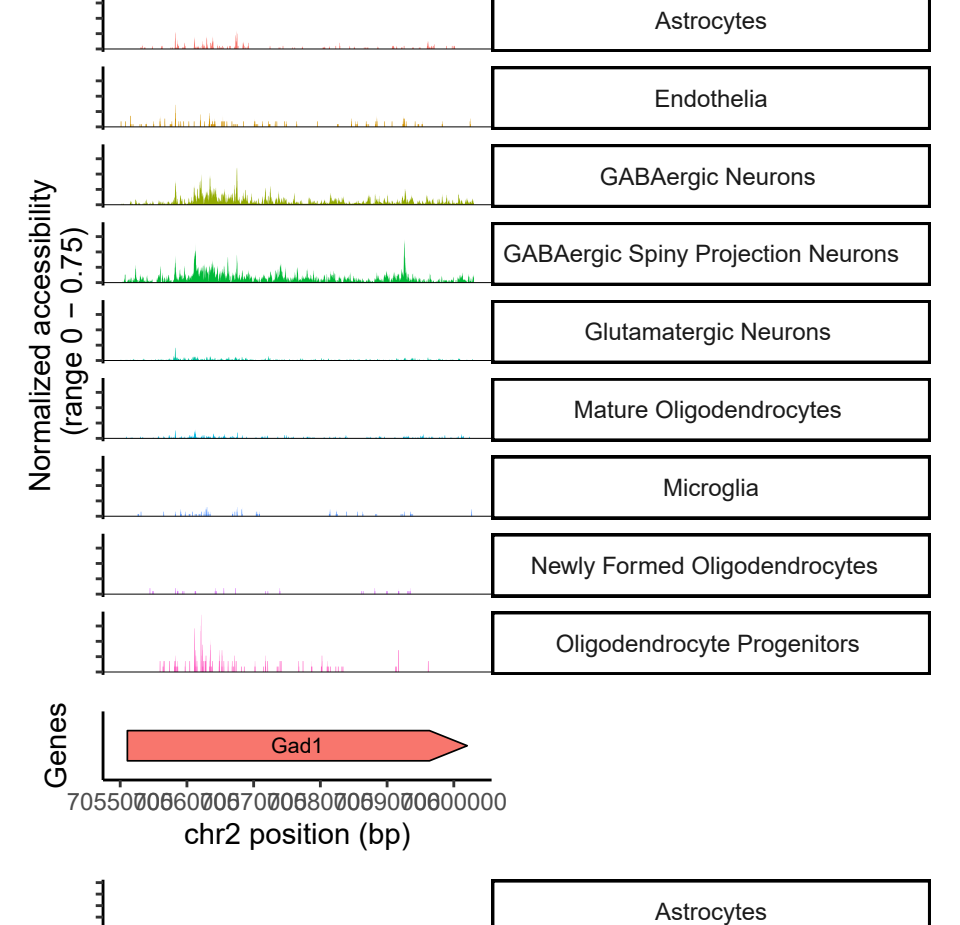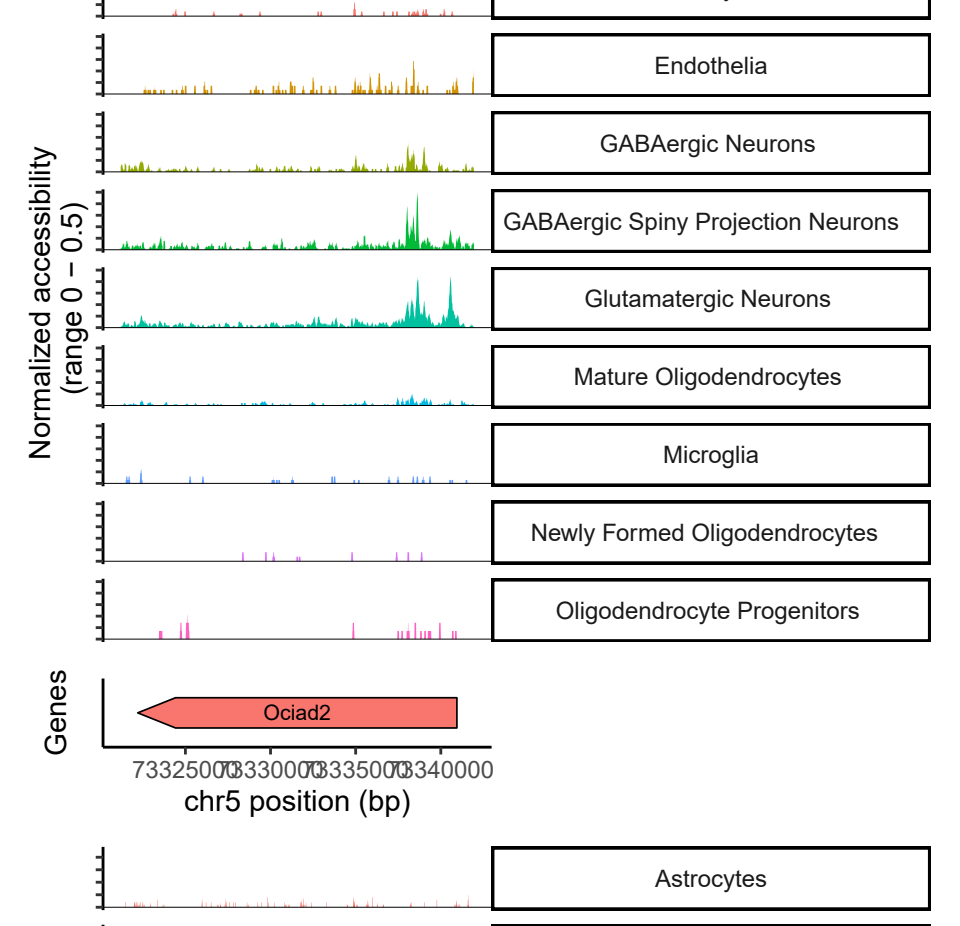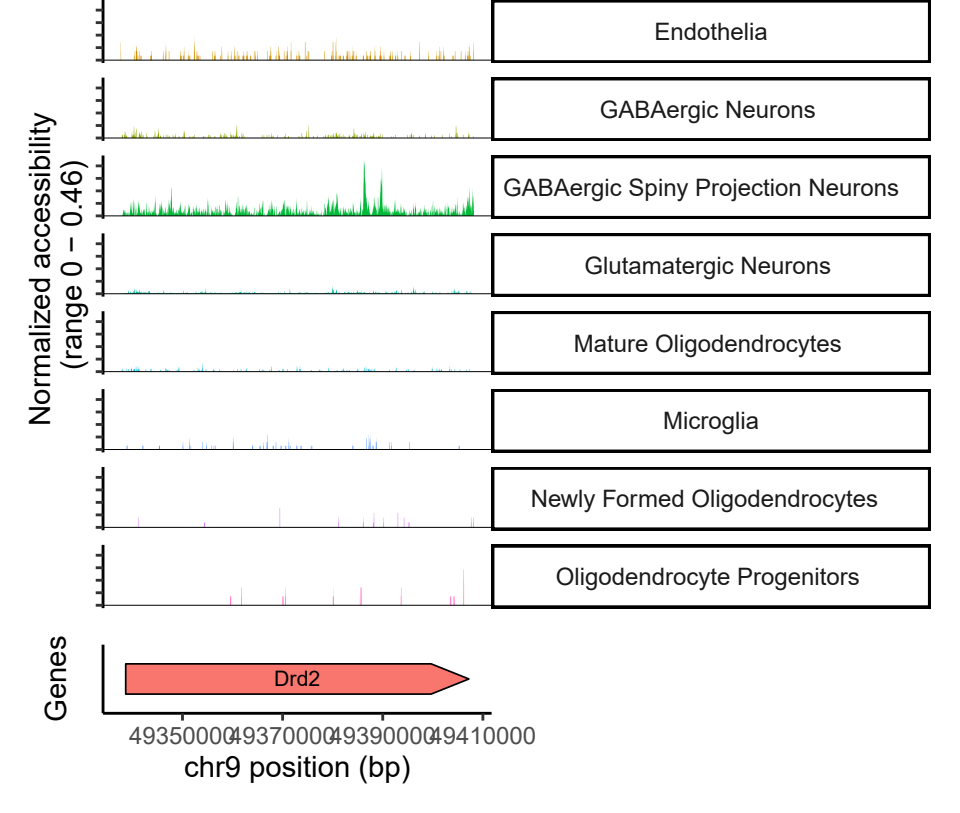

Supplement: Supplementary file 3 — Supplementary Data 1 [file 41467_2021_21515_MOESM3_ESM.zip › Corrected_CelltypeMarker_Plots/MouseSSpCortex.markeraccessibility/MouseSSpCortex.MSN.markeraccessibility.pdf]

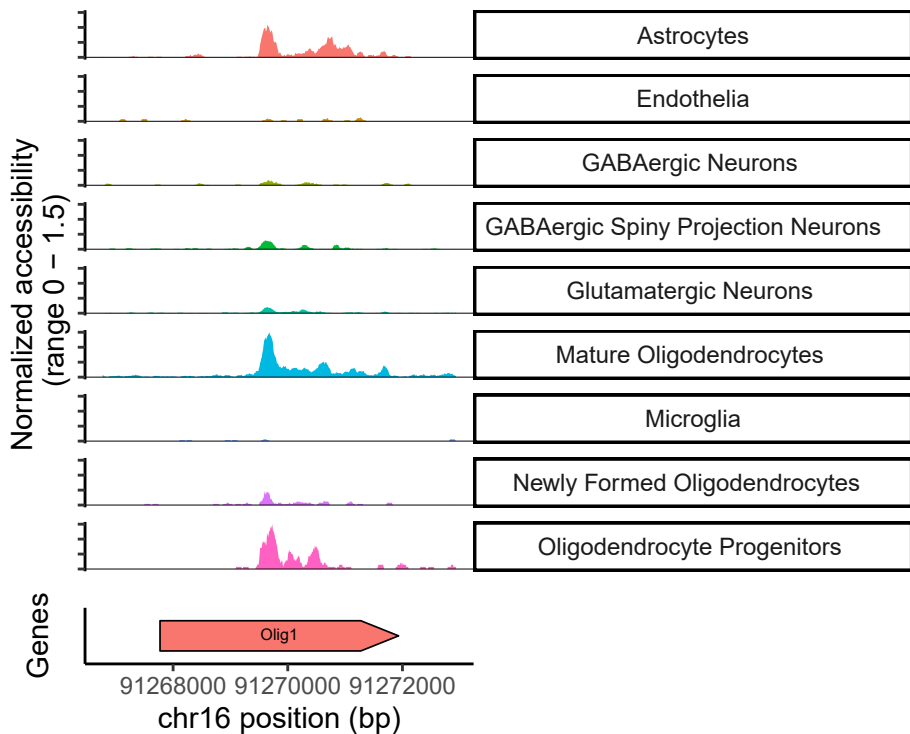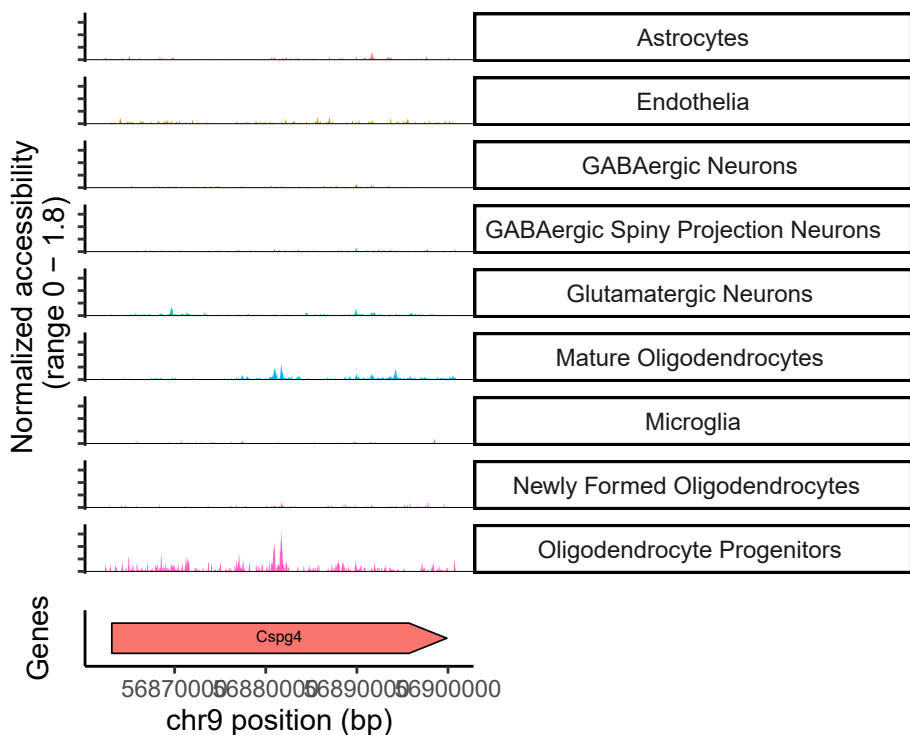

Supplement: Supplementary file 3 — Supplementary Data 1 [file 41467_2021_21515_MOESM3_ESM.zip › Corrected_CelltypeMarker_Plots/MouseSSpCortex.markeraccessibility/MouseSSpCortex.OPC.markeraccessibility.pdf]

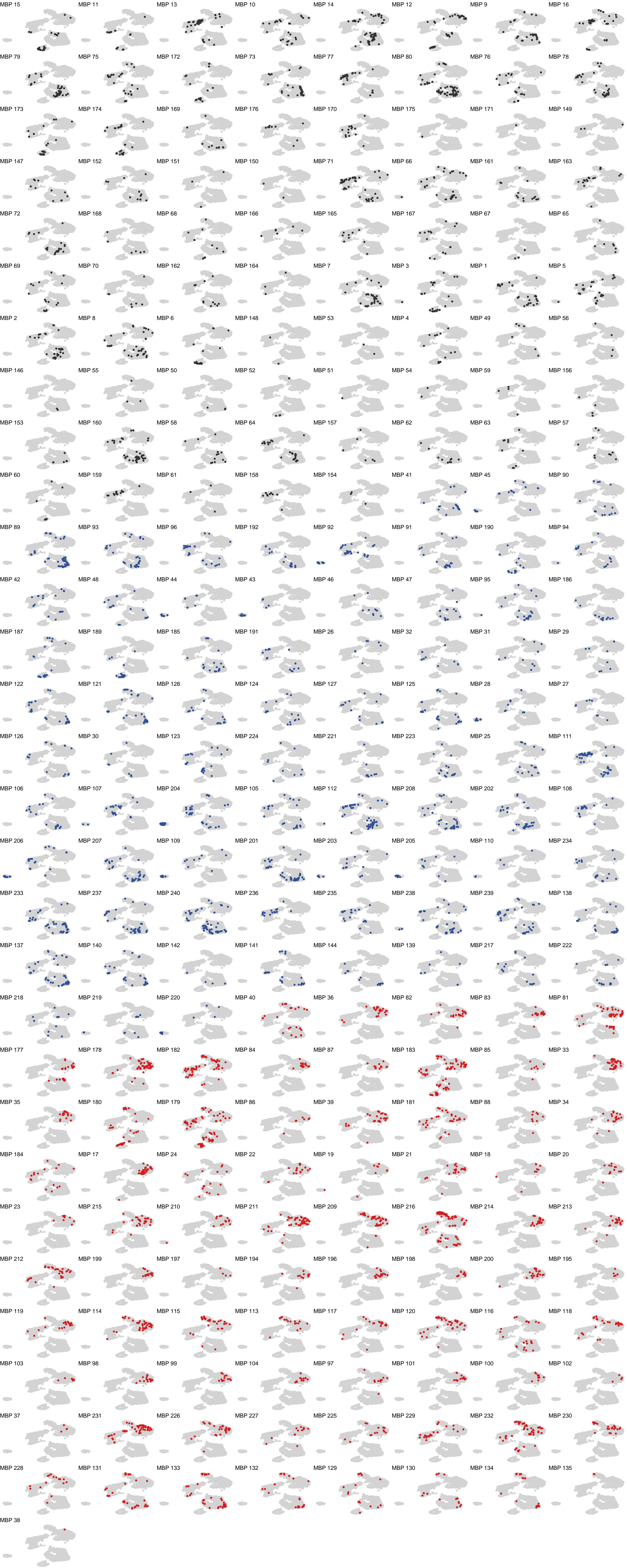

Supplement: Supplementary file 4 — Supplementary Data 2 [file 41467_2021_21515_MOESM4_ESM.zip › CerebralIschemia.punches.UMAP.pdf]

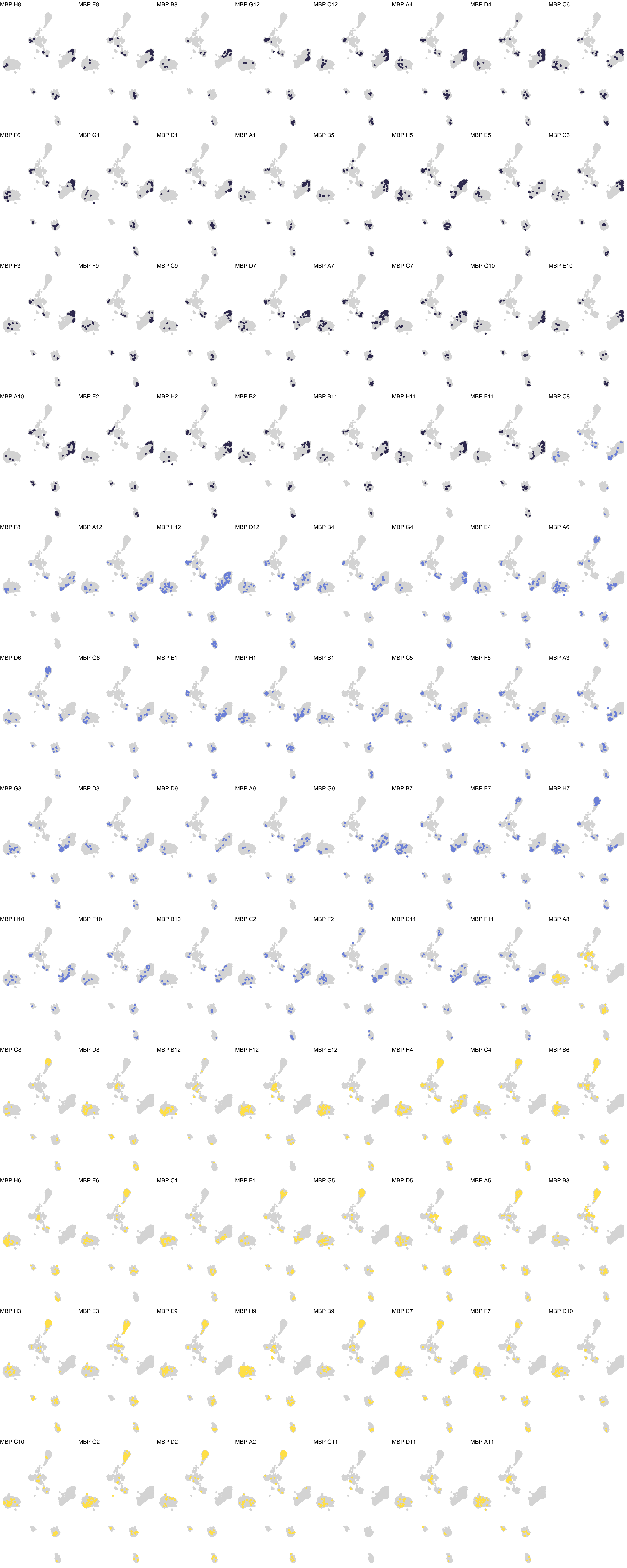

Supplement: Supplementary file 4 — Supplementary Data 2 [file 41467_2021_21515_MOESM4_ESM.zip › SSpCortex.punches.UMAP.pdf]

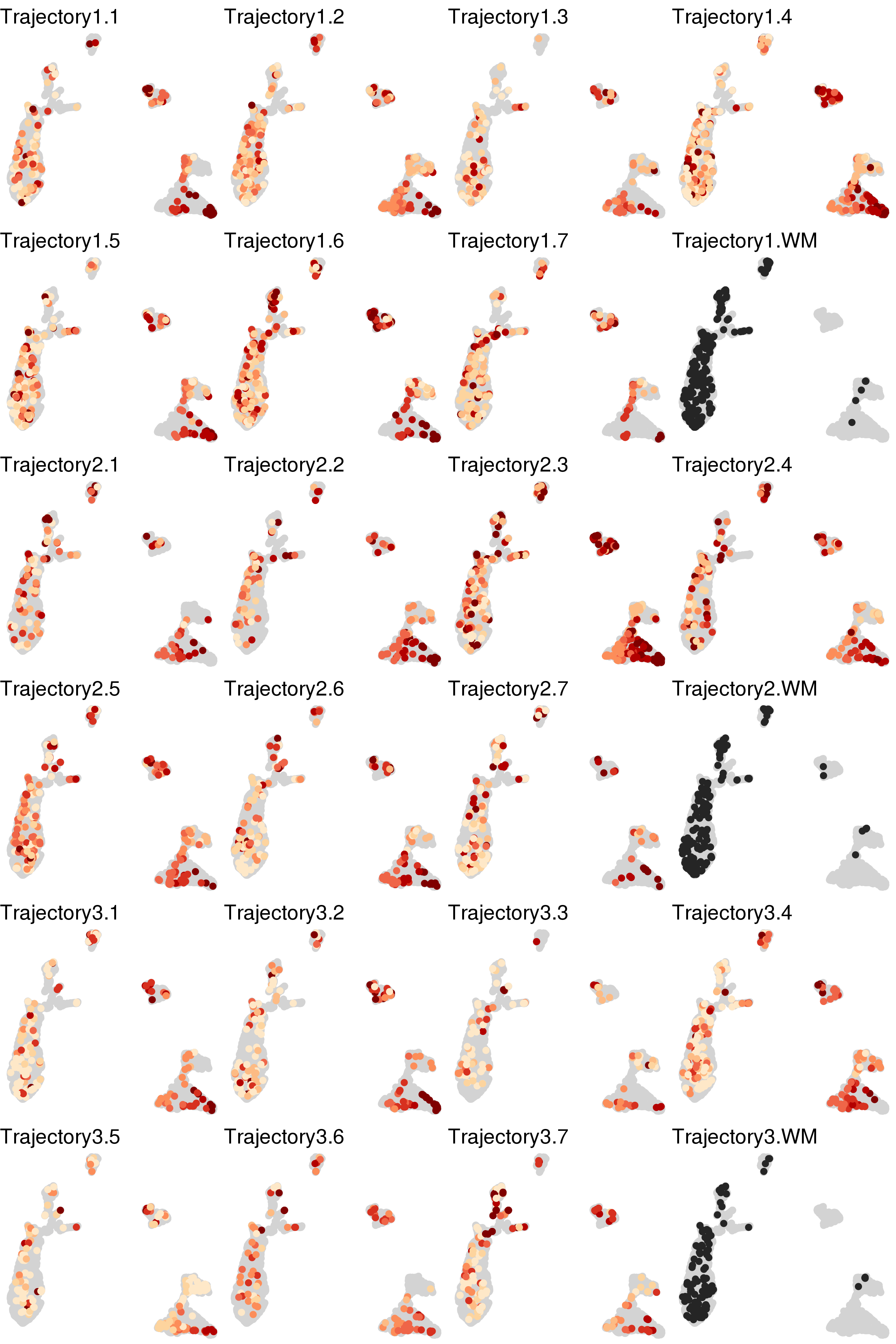

Supplement: Supplementary file 4 — Supplementary Data 2 [file 41467_2021_21515_MOESM4_ESM.zip › HumanVISp.trajectories.UMAP.png]
